# Acid sphingomyelinase recruits palmitoylated CD36 to membrane rafts and enhances lipid uptake

**DOI:** 10.1016/j.jbc.2025.110213

**Published:** 2025-05-08

**Authors:** Meng Ding, Yun Zhang, Xiaoting Xu, Yuan Zhu, Hui He, Tianyu Jiang, Yashuang Huang, Wenfeng Yu, Hailong Ou

**Affiliations:** Department of Biochemistry and Molecular Biology, School of Basic Medicine, Guizhou Medical University, Gui'an, Guizhou, China

**Keywords:** CD36, palmitoylation, acid sphingomyelinase, foam cell, lipid raft

## Abstract

CD36 palmitoylation increases its membrane localization and is required for CD36-mediated uptake of oxidized low-density lipoprotein (oxLDL). Acid sphingomyelinase (ASMase) is transported to the plasma membrane, where it promotes lipid raft clustering, facilitating membrane protein anchoring for biological functions. We investigated the effects of oxLDL on CD36 palmitoylation and explored the role of ASMase in CD36 membrane translocation. We found that oxLDL increased CD36 palmitoylation and drives its intracellular trafficking from the endoplasmic reticulum to the plasma membrane lipid rafts in macrophages. Affinity purification followed by mass spectrometry analysis identified CD36 bound to ASMase in the plasma membrane. The CD36/ASMase binding was enhanced by oxLDL treatment. Genetic ablation and pharmacological inhibition of ASMase reduced CD36 recruitment to lipid rafts and inhibited CD36 intracellular signaling and lipid uptake. Moreover, inhibiting Sortilin to block ASMase intracellular trafficking and reduce membrane ASMase also caused a sharp decrease in the amount of membrane CD36. In addition, ASMase overexpression dramatically promoted palmitoylated CD36 membrane localization but not CD36 without palmitoylation, in which the modification was inhibited by 2-bromopalmitate (2-BP) treatment or point mutation at the palmitoylation site. Moreover, ASMase knockout inhibited CD36 membrane recruitment both in peritoneal macrophages and in the aorta, and attenuated lipid accumulation in atherosclerotic plaques in mice. Finally, we found oxLDL activated extracellular signal-regulated kinase1/2 (ERK1/2)/specificity protein (SP1) signaling, upregulating ASMase transcription and promoting sphingomyelin catabolism. Therefore, these data demonstrate that ASMase expression induced by oxLDL is required for palmitoylated CD36 membrane translocation during foam cell formation in macrophages.

Sphingomyelin (SM) is the most abundant sphingolipid in mammalian cells, primarily distributed in the outer leaflet of the plasma membrane. Besides being a structural component of the cell membrane, SM acts as a bioactive lipid and can be hydrolyzed by sphingomyelinase (SMase) at phosphodiester bonds to generate phosphocholine (PC) and ceramide (1). The latter is further degraded into sphingosine and sphingosine-1-phosphate (S1P) (1). Acid sphingomyelinase (ASMase), a key enzyme in SM catabolism, is encoded by *Smpd1*. Two isoforms of ASMase have been identified through differential trafficking and post-translational: lysosomal resident form (L-ASMase) and secretory ASMase (S-ASMase), with the latter distributed in the extracellular leaflets of the plasma membrane (2).

Lipid rafts (LRs) are specialized membrane microdomains or detergent-resistant membranes, characterized by their insolubility in Triton X-100 extraction. LRs are enriched in cholesterol and lipids with saturated acyl chains, including sphingolipids and glycosphingolipids, and contain proteins, such as flotillins and caveolin 1 (Cav-1). Highly ordered, dynamic lipid structures serve as platforms for the recruitment, organization, and interaction of specific proteins, especially transmembrane and intracellular signaling proteins, and are involved in processes like signal transduction, trafficking, lipid transport, and cytoskeletal rearrangements (3, 4, 5). L-ASMase is transported to the cellular membrane by lysosomal exocytosis, and then the S-ASMase cleaves its substrate, SM, to generate ceramide upon stimulation (6, 7). ASMase membrane accumulation and ceramide production promote LR clustering, enhancing LR functions (6, 7).

Circulating monocytes infiltrate the endothelium of the arterial wall, where they differentiate into macrophages to eliminate the accumulated oxidized low-density lipoprotein (oxLDL). Excessive oxLDL uptake by macrophages transforms them into foam cells, initiating atherosclerotic lesion progression (8). ASMase, abundantly expressed in macrophages, accelerates atherosclerosis by promoting LDL aggregation, binding of LDL to intimal proteoglycans and other extracellular matrix proteins, thus causing subendothelial retention (9). ASMase-induced LDL aggregation and retention enhance macrophage LDL uptake and cholesterol esterification, driving foam cell formation (9).

CD36, a member of the class B scavenger receptor family, is responsible for oxLDL uptake by macrophages. It is expressed on the cell surface with two short N- and C-terminal intracellular tails and two transmembrane domains separated by a large extracellular loop that is heavily glycosylated (10). CD36 undergoes palmitoylation at four cytosolic cysteine residues, Cys3 and Cys7 in the N-terminus, and Cys464 and Cys466 in the C-terminus, which are required for proper intracellular trafficking to the membrane LR (10). When excess oxLDL is deposited on the arterial wall, CD36 binds to and internalizes it in macrophages, promoting foam cell formation. The CD36 palmitoylation and LR translocation are increased by oxidized high-density lipoproteins (11). In addition, cellular lipid uptake by CD36 requires the association of other membrane molecules such as platelet-activating factor, selenoprotein K, and Cav-1 (12, 13, 14). On the other hand, ASMase has been shown to undergo membrane clustering upon oxLDL stimulation and is involved in promoting the intracellular trafficking and subcellular localization of palmitoylated proteins from the Golgi to the plasma membrane (15). Therefore, we here attempted to elucidate the role of ASMase in CD36-mediated lipid uptake. We provide evidence that oxLDL increases CD36 palmitoylation and upregulates ASMase expression, promoting SM hydrolysis and facilitating the palmitoylated CD36 recruitment to LRs, thereby increasing lipid uptake.

## Results

### OxLDL increases CD36 palmitoylation and intracellular trafficking

**Figure 1:**
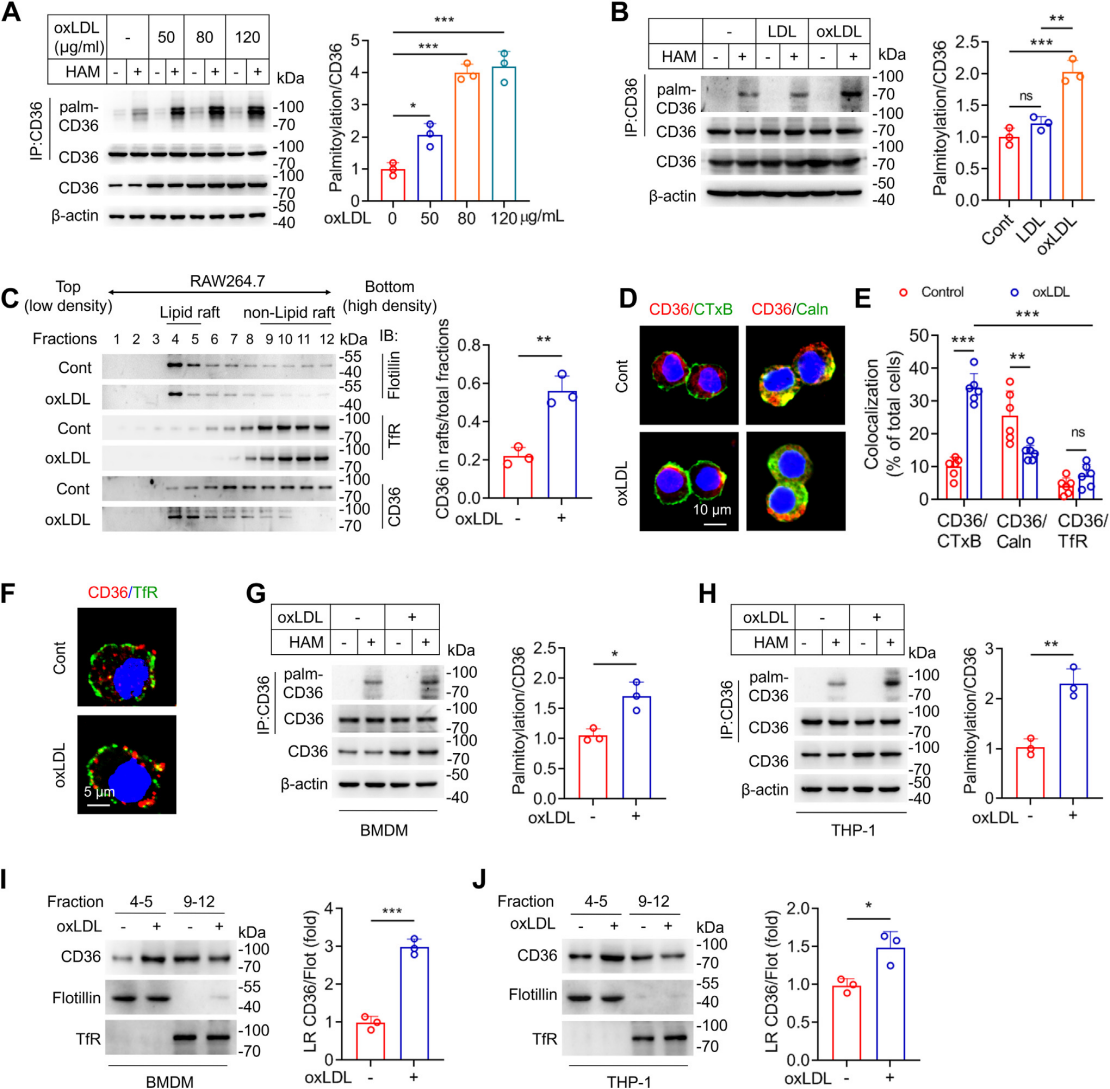
**OxLDL increases CD36 palmitoylation and membrane translocation.***A*, RAW264.7 macrophages are incubated with the indicated concentrations of oxLDL for 24 h. CD36 palmitoylation is determined using the acyl biotin exchange (ABE) method. HAM, hydroxylamine. *B*, CD36 palmitoylation levels in cells treated with LDL (50 μg/ml), oxLDL (50 μg/ml) for 24 h. *C*, subcellular fractions are separated by discontinuous sucrose gradient ultracentrifugation in cells incubated with or without 50 μg/ml oxLDL. CD36 distribution is detected by Western blot. Fractions 4 and 5, enriched in flotillin-1, contain lipid rafts (LR). Transferrin receptor (TfR) was used as non-raft marker. *D*, immunofluorescence staining shows CD36 (red) and CTxB (green) in left; CD36 (*red*) and Caln (Calnexin, *green*) in right. *E*, colocalization of CD36 with CTxB, Caln and TfR. Data were from six separated fields with three repeats. *F*, immunofluorescence staining shows CD36 (*red*) and TfR (*green*). *G* and *H*, CD36 palmitoylation in oxLDL-treated BMDMs and human THP-1 cells. *I* and *J*, subcellular location of CD36 in oxLDL-treated BMDMs and human THP-1 cells. Data are analyzed by one-way ANOVA followed by Tukey’s *post hoc* test in (*A* and *B*) (n = 3), by Student's *t* test in (*C*, *G*–*J*) (n = 3), and by two-way ANOVA in (*E*) (n = 6). Mean ± SD, ∗*p* < 0.05, ∗∗*p* < 0.01, ∗∗∗*p* < 0.001. ns, not significant.

RAW264.7 cells were incubated with various concentrations of oxLDL for 24 h. CD36 palmitoylation levels dramatically increased with oxLDL treatment at concentrations of 50, 80, and 120 μg/ml (Fig. 1*A*). The modification was not significantly changed in native LDL-treated cells (Fig. 1*B*). Cells were fractionated using discontinuous sucrose density gradient centrifugation (5–40%). Immunoblotting showed that CD36 was evenly distributed across fractions 7 to 12 in control cells, but increased recruitment to the LR regions was observed in fractions 4 and 5, as evidenced by the presence of the flotillin-1 marker after oxLDL exposure (Fig. 1*C*). Cholera toxin B (CTxB) specifically binds to GM1 ganglioside that is exclusively found in rafts. Immunofluoresence staining showed that oxLDL reduced the localization of CD36 in endoplasmic reticulum (ER) and increased the movement from the plasma to FITC-CTxB-labeled LRs but not nonraft region (Fig. 1, *D*–*F*). Caveolae is a specific type of membrane LR formed by caveolins and were also found co-localization with CD36 upon oxLDL treatment detected by immunofluoresence staining and immunoprecipitation (Fig. S1, *A* and *B*). Finally, the CD36 palmitoylation and membrane localization were increased in oxLDL-treated bone marrow-derived macrophage (BMDM) and human THP-1 cells (Fig. 1, *G*–*J*). Together, these results demonstrate that oxLDL increases CD36 palmitoylation and membrane translocation from the ER in macrophages.

### ASMase associates with CD36 in membranes of oxLDL-induced macrophages

**Figure 2:**
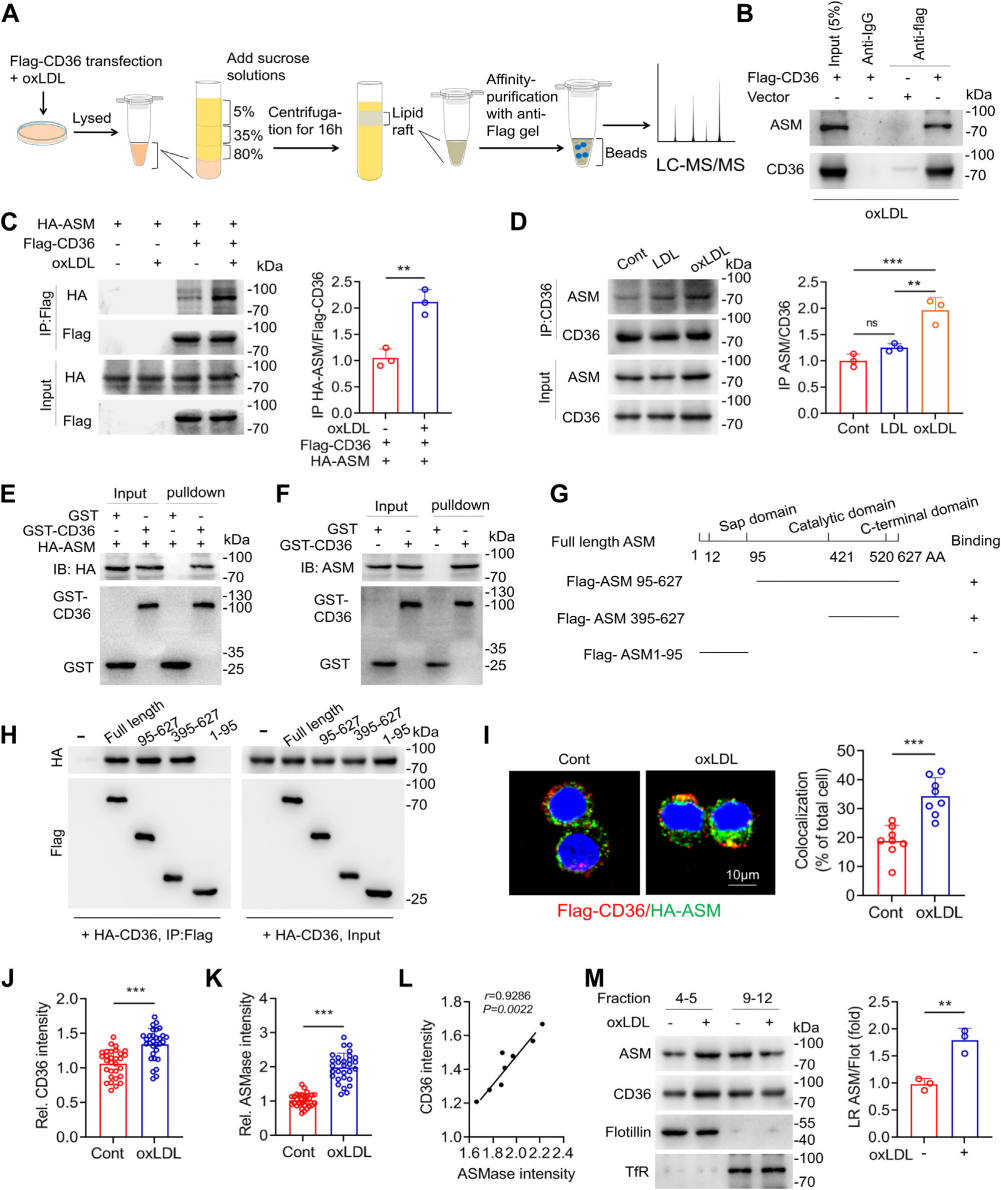
**CD36 interacts with ASMase in membrane of oxLDL-induced cells.***A*, schematic representation of the steps for identification of CD36-associated proteins in the membrane. RAW264.7 cells transfected with Flag-CD36 are incubated with oxLDL (50 μg/ml). Plasma LR are isolated, and CD36-associated proteins are purified using an anti-Flag affinity column. The protein complex is analyzed by mass spectrometry. *B*, immunoblotting analysis of presence of ASMase in eluates immunoprecipitated with anti-Flag-CD36 antibodies. Blank vector used as control. *C*, cells transfected with Flag-CD36 and HA-ASM undergo immunoprecipitation with anti-Flag agarose gel, followed by immunoblotting with CD36 and ASMase antibodies. *D*, co-immunoprecipitation assay shows the association of endogenous CD36 and ASMase. *E* and *F*, purified GST-CD36 is incubated with HA-ASM and cell lysates, and the complexes are subjected to a pull-down assay. *G* and *H*, schematic diagram of full-length and deletion mutants of ASMase, and the domain responsible for interaction with CD36 was identified by using co-immunoprecipitation. *I*, immunofluorescence staining shows Flag-CD36 (*red*) and HA-ASM (*green*). Percentages of Flag-CD36/HA-ASM colocalization cells in total cells were from eight separated fields. *J* and *K*, Flag-CD36 and HA-ASM intensities in randomly selected 30 cells. *L*, Spearman analysis for the relationship between CD36 and ASMase intensities. *M*, cell lysate were centrifugated and fractionized. The ASMase in the raft and nonraft fractions were detected by Western blot. Representative blots and images were from three independent experiments. Data are analyzed by Student's *t* test in (*C*, *I*–*K*, *M*), by one-way ANOVA followed by Tukey’s *post hoc* test in (*D*) and by Spearman Correlation Analysis in (*L*), mean ± SD, n = 3. ∗*p* < 0.05, ∗∗*p* < 0.01, ∗∗∗*p* < 0.001.

RAW264.7 cells were transfected with Flag-CD36 and treated with oxLDL. After isolating membrane LRs by ultracentrifugation, Flag-CD36 was affinity-purified, and the binding proteins were subjected to MS (Fig. 2*A*). MS analysis revealed ASMase as a potentially CD36-binding partner. The presence of ASMase in eluates was confirmed by immunoblotting (Fig. 2*B*). Co-immunoprecipitation experiments revealed that CD36 was associated with ASMase in oxLDL-treated RAW264.7 cells (Figs. 2, *C* and *D*, S2*A*), and showed the binding was significantly increased in comparison to untreated and LDL-treated cells (Fig. 2*D*). The association of CD36/ASMase was also detected in BMDM and THP-1 after oxLDL stimulation (Fig. S2, *B* and *C*). Purified glutathione S-transferase (GST)-fused CD36 protein was pulled down with ASMase, either from recombinant ASMase or cell extracts, suggesting the *in vitro* interaction of CD36 and ASMase in RAW264.7 cells (Fig. 2, *E* and *F*). ASMase contains an N-saposin B-type domain, intermediate catalytic sites, and a C-domain. Accordingly, we explored the specific region responsible for this interaction. OxLDL-treated cells were transfected with hemagglutinin (HA)-CD36 and vectors expressing full-length or truncated ASMase. CD36 co-immunoprecipitated with full-length, catalytic, and C-terminal domains, but not with the truncated mutant containing the Sap domain, suggesting that the interaction was mediated by the C-terminal domain (Fig. 2, *G* and *H*). Immunofluorescence staining of cells transfected with CD36 and ASMase vectors showed increased co-localization of the two proteins in the membrane upon oxLDL treatment (Fig. 2*I*). OxLDL increased membrane CD36 and ASMase, and the CD36 expression was positively correlated to ASMase (Fig. 2, *J*–*L*). The enrichment of ASMase in plasma LR was further confirmed by Western blots (Figs. 2*M*, S3, *A* and B). These data demonstrate that CD36 and ASMase form a complex within LRs in oxLDL-treated cells.

### ASMase is required for palmitoylated CD36 LR recruitment during foam cell formation

**Figure 3:**
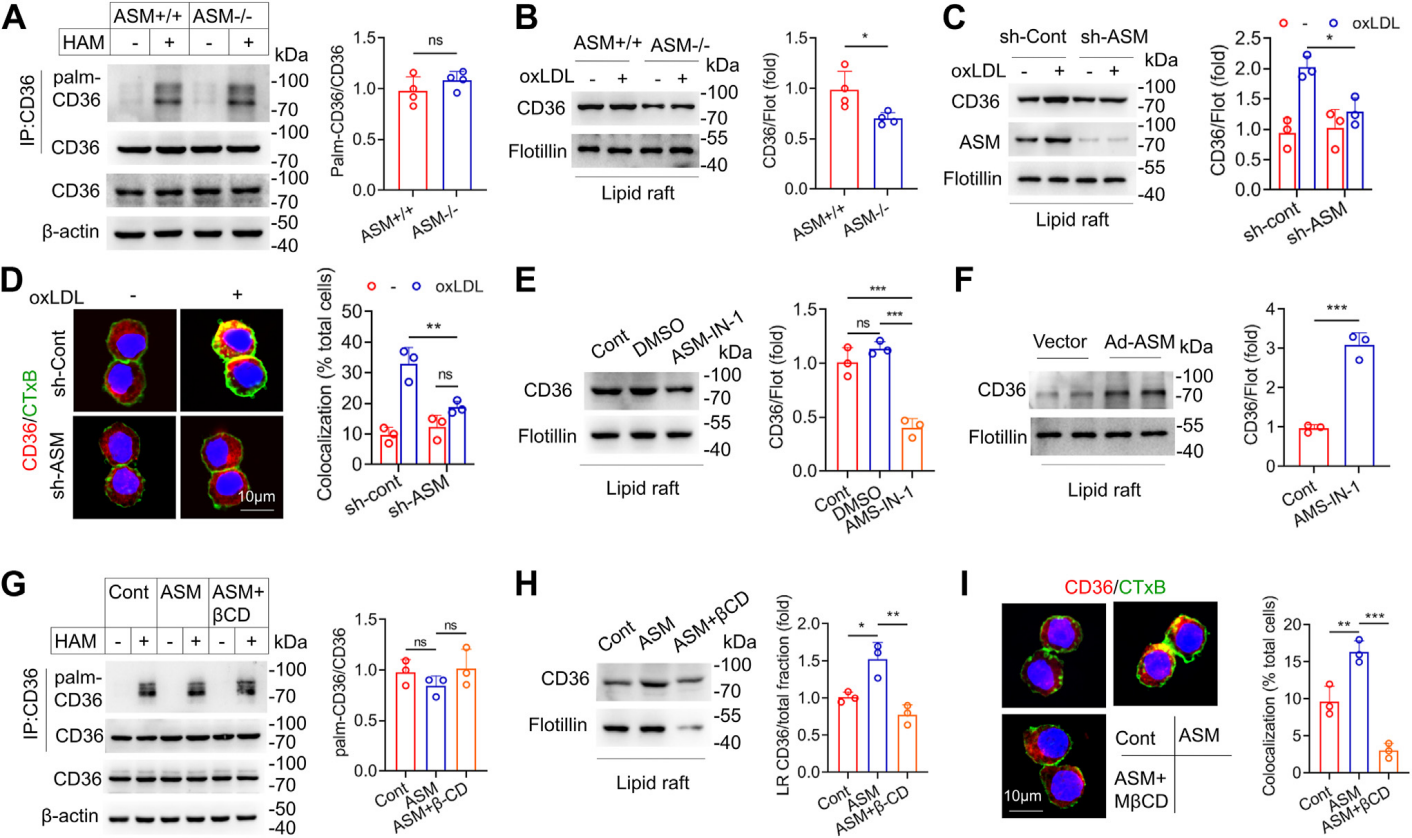
**ASMase promotes lipid recruitment of palmitoylated CD36 during foam cell formation.***A*, the ABE assay measures CD36 palmitoylation in oxLDL-incubated peritoneal macrophages isolated from wild-type and ASMase−/− mice (Student's *t* test, n = 6 mice per group). *B*, LR localization of CD36 in wild-type and ASMase deficient macrophages (Student's *t* test, n = 6 mice per group). *C*, LR localization of CD36 in RAW264.7 macrophages with ASMase knockdown *via* shRNA (two-way ANOVA with Tukey’s test, n = 3). *D*, RAW264.7 macrophages are stained with anti-CD36 antibody (*red*) and FITC-CTxB (*green*) (two-way ANOVA with Tukey’s test, n = 3). *E*, Western blot analysis of CD36 localization in LR in oxLDL-treated cells treated with ASM-IN-1 (3 μM), an ASMase inhibitor, for 24 h. (one-way ANOVA with Tukey’s test, n = 3). *F*, Western blot analysis of CD36 localization in LR in oxLDL-treated cells transfected with adenovirus-mediated ASMase expressing vector. (Student's *t* test, n = 3). *G* and *H*, CD36 palmitoylation and LR localization in oxLDL-treated cells treated with recombinant ASMase (20 μg/ml) for 24 h and with or without MβCD (5 mmol/L) for 2 h. (one-way ANOVA with Tukey’s test, n = 3). *I*, immunofluorescence staining shows CD36 (*red*) and FITC-CTxB (*green*) in oxLDL-treated cells treated with ASMase and ASMase + MβCD (one-way ANOVA with Tukey’s test, n = 3). ∗*p* < 0.05, ∗∗*p* < 0.01, ∗∗∗*p* < 0.001. ns, not significant.

Primary mouse peritoneal macrophages were isolated from ASMase-deficient (ASM−/−) and wild-type (ASM+/+) mice and incubated with oxLDL. The level of CD36 palmitoylation was not significantly different between ASM−/− and ASM+/+ macrophages (Fig. 3*A*). However, LR recruitment of CD36 was substantially reduced in ASM−/− macrophages (Fig. 3*B*). Knockdown of ASMase using short hairpin RNA (shRNA) in both RAW264.7 and THP-1 cells similarly reduced oxLDL-induced CD36 recruitment to LRs compared to sh-control cells, which corroborated the results in ASMase-knockout macrophages (Figs. 3, *C* and *D* and S4). The LR recruitment of CD36 was also inhibited by the ASMase pharmacological inhibitor, ASM-IN-1 (Figs. 3*E* and S5). Conversely, ASMase overexpression induced CD36 membrane localization (Fig. 3*F*). OxLDL promotes ASMase aggregation and LR clustering, providing a large and stable platform for protein anchoring and transmembrane signal transduction (16). Accordingly, we elucidated the role of LR clustering in ASMase-mediated CD36 membrane translocation by using methyl-β-cyclodextrin (MβCD), which effectively removes cholesterol from cell membranes, to disrupt the LR structure. MβCD treatment did not change the CD36 palmitoylation, but reduced ASMase-induced CD36 recruitment to LRs (Fig. 3, *G*–*I*). These data suggest that palmitoylated CD36 LR recruitment requires ASMase and depends on membrane integrity and LR clustering.

### ASMase increases CD36 activity during foam cell formation

**Figure 4:**
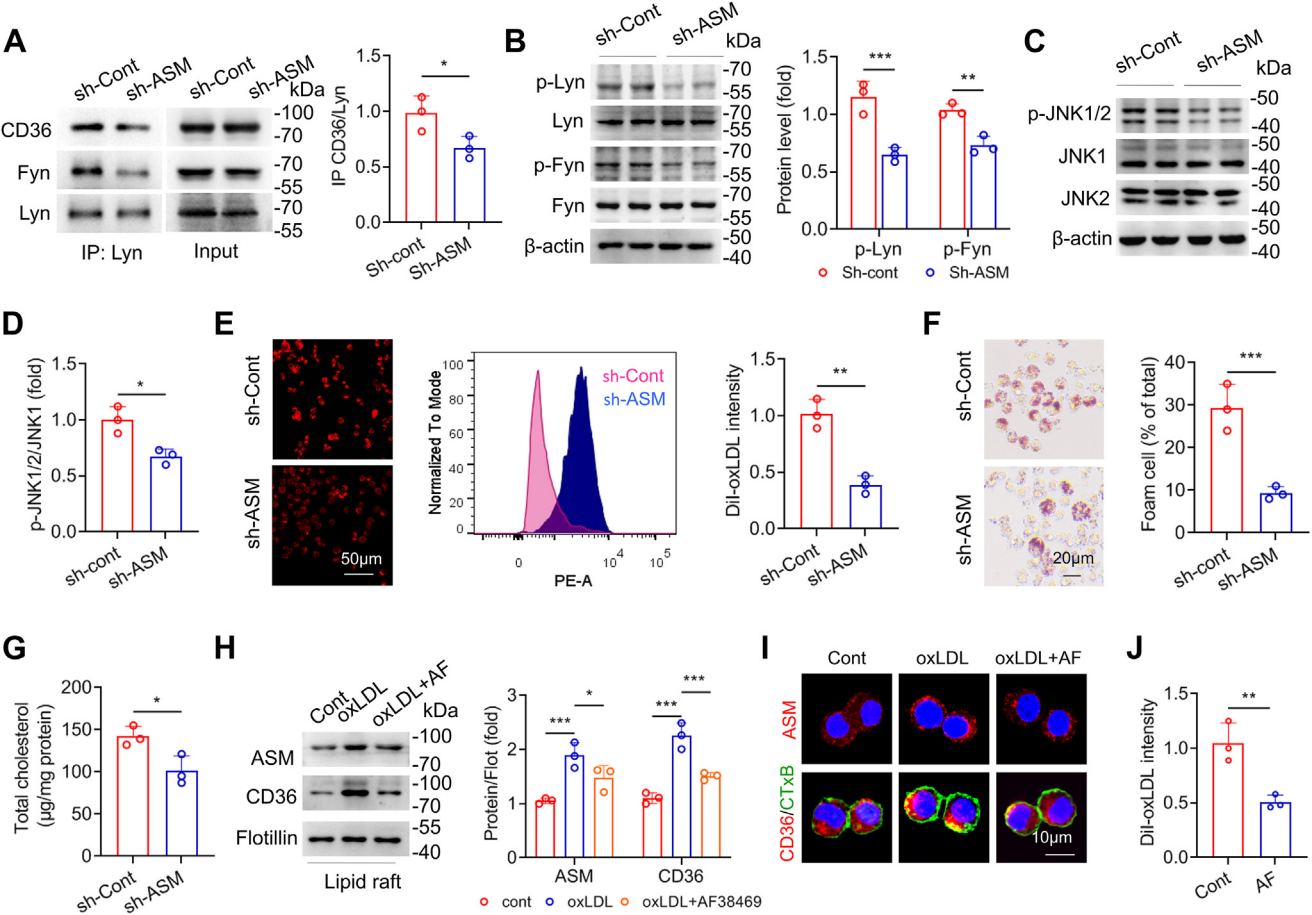
**Reduced membrane AMSase inhibits CD36-mediated downstream signaling in RAW264.7 macrophages.***A*, co-immunoprecipitation assay measures CD36/Fyn/Lyn association in cells with ASMase knockdown. Cells are transfected with sh-ASMase and incubated with oxLDL for 24 h (Student's *t* test, n = 3). *B*–*D*, phosphorylation levels of Fyn, Lyn, and JNK1/2 are analyzed in oxLDL-treated cells with ASMase knockdown (Student's *t* test, n = 3). *E*, cells transfected with sh-ASMase are incubated with DiI-oxLDL (30 μg/ml) for 6 h. DiI-oxLDL uptake is measured by flow cytometry (Student's *t* test, n = 3). *F*, cells transfected with sh-ASMase are incubated with oxLDL for 24 h and stained with Oil Red O (Student's *t* test, n = 3). *G*, total cholesterol is measured in cells transfected with sh-ASMase and incubated with oxLDL (Student's *t* test, n = 3). *H*, LR localization of CD36 and ASMase is analyzed in cells incubated with oxLDL with or without Sortilin inhibitor AF38469 (one-way ANOVA followed by Tukey’s *post hoc* test, n = 3). *I*, cells incubated with oxLDL with or without AF38469 are stained with anti-CD36 antibody (*red*) and FITC-CTxB (*green*). *J*, DiI-oxLDL uptake is measured in cells incubated with or without AF38469 (Student's *t* test, n = 3). ∗*p* < 0.05, ∗∗*p* < 0.01, ∗∗∗*p* < 0.001.

CD36 translocates to membranes where it interacts with Lyn and Fyn kinases, inducing downstream signaling, especially by increasing c-Jun N-terminal kinase (JNK) activity. RAW264.7 cells treated with ASMase-targeting shRNA and incubated with oxLDL. Co-immunoprecipitation results showed that ASMase knockdown reduced the association of CD36 with Lyn and Fyn (Fig. 4*A*). The phosphorylation levels of Lyn, Fyn, and JNK1/2 were significantly reduced following ASMase knockdown (Fig. 4, *B*–*D*). ASMase knockdown significantly reduced DiI-oxLDL uptake, foam cell formation, and intracellular cholesterol accumulation (Fig. 4, *E*–*G*). In addition, we transfected macrophages with adenovirus-mediated ASMase and found the overexpressing ASMase increased DiI-oxLDL uptake, accelerating foam cell formation and cholesterol accumulation (Fig. S6, *A*–*C*).

We investigated whether inhibiting ASMase subcellular localization could reduce CD36 membrane translocation. Sortilin mediates intracellular ASMase trafficking (17, 18). RAW264.7 cells were then incubated with oxLDL in the presence or absence of AF38469, a pharmacological inhibitor of Sortilin, and membrane LRs were isolated. As expected, immunoblotting results showed that oxLDL-induced ASMase accumulation in the membrane was reduced by AF38469 (Fig. 4*H*). Importantly, the inhibition of ASMase intracellular trafficking by AF38469 also reduced the translocation of CD36 in oxLDL-treated cells (Fig. 4*H*). When LRs were labeled with FITC-CTxB, a reduction in CD36 translocation was observed, evidenced by the decreased co-localization of CTxB (green) and CD36 (red) (Fig. 4*I*). OxLDL uptake was reduced following AF38469 treatment (Fig. 4*J*). Finally, endoplasmic reticulum (ER) stress causes proteins retention in the ER. However, we found the ER stress markers such as GRP78, p-PERK and CHOP levels were not significantly increased in sh-ASM-treated cells and ASM−/− cells, even showing reductions upon oxLDL stimulation (Fig. S7, *A* and *B*), which suggested the ASMase-modulated CD36 membrane translocation was independent on ER stress. Therefore, these findings demonstrated that membrane ASMase facilitates CD36 LR recruitment and activity during foam cell formation.

### ASMase does not promote membrane recruitment of non-palmitoylated CD36

**Figure 5:**
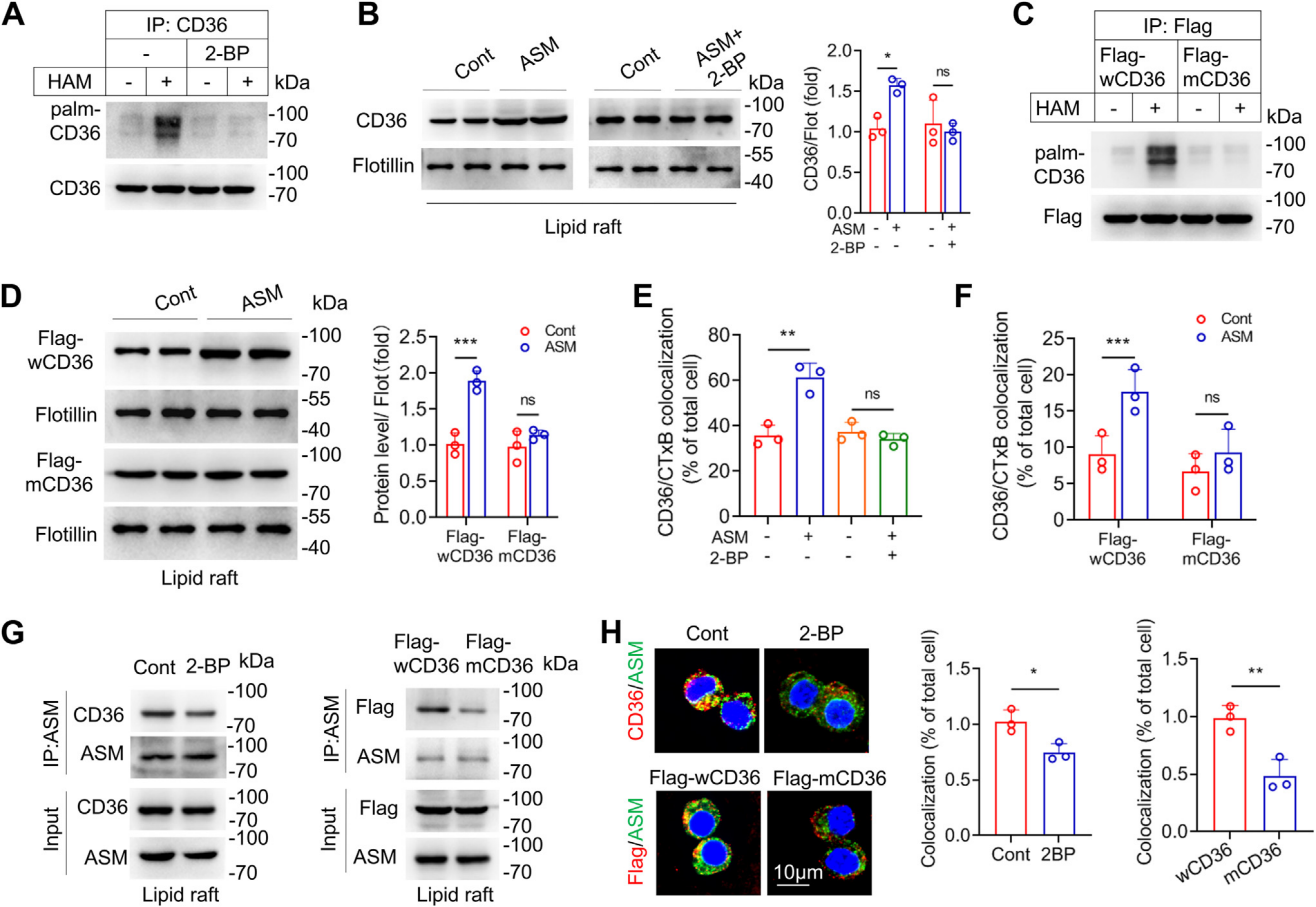
**Reduced CD36 palmitoylation inhibits ASMase-mediated CD36 LR localization.***A*, CD36 palmitoylation is analyzed in cells treated with oxLDL and with or without 2-BP. *B*, CD36 LR localization is measured in oxLDL-incubated cells treated with ASMase or ASMase + 2-BP. *C*, CD36 palmitoylation is analyzed in oxLDL-induced cells transfected with Flag-wCD36 (wild-type) or Flag-mCD36 (mutant). *D*, CD36 LR localization is measured in cells transfected with Flag-wCD36 or Flag-mCD36, following oxLDL incubation. *E* and *F*, RAW264.7 macrophages were stimulated with oxLDL and treated as indicated. The cells were then stained with CD36 and CTxB, and percentage of cells with CD36/CTxB co-localization in total cells were counted. *G*, inhibited association of ASMase and CD36 in LR after CD36 palmitoylation were blocked by 2-BP (*left*) or transfection with mCD36 (*right*) in oxLDL-treated cells. *H*, immunofluorescence staining assay shows reduced co-localization of ASMase with CD36 of palmitoylation inhibited by 2-BP or mutation in oxLDL-treated cells. CD36 appears red and ASMase appears green. All data are analyzed by Student's *t* test, mean ± SD, n = 3. ∗*p* < 0.05, ∗∗*p* < 0.01, ∗∗∗*p* < 0.001.

To investigate whether ASMase promotes membrane recruitment of non-palmitoylated CD36, we used 2-bromopalmitate (2-BP), an inhibitor of palmitoyl acyl transferase, to block palmitoylation, and found a reduced oxLDL-induced CD36 palmitoylation by 2-BP (Fig. 5*A*). The 2-BP treatment also attenuated the ASMase-induced CD36 membrane recruitment, suggesting that ASMase does not promote the membrane translocation of non-palmitoylated CD36 (Fig. 5*B*). We further reduced CD36 palmitoylation by mutating four conserved cysteine residues involved in palmitoylation at the NH_2_ (Cys3 and Cys7) and COOH termini (Cys464 and Cys466). The mutated CD36 (mCD36) and wild-type CD36 (wCD36) were transfected into RAW264.7 cells. Palmitoylation of mCD36 was barely detectable after oxLDL stimulation (Fig. 5*C*). Consistent with the results of 2-BP treatment, ASMase increased the membrane recruitment of wCD36 but not mCD36 in response to oxLDL stimulation (Fig. 5*D*). Immunofluorescence results further confirmed that inhibiting CD36 palmitoylation, either by 2-BP or mutation, reduced CTxB/CD36 co-localization in oxLDL-treated cells (Fig. 5, *E* and *F*). Moreover, 2-BP treatment or mutation at palmitoylation sites reduced the association between CD36 and ASMase in the membranes of oxLDL-treated cells (Fig. 5, *G* and *H*). These data demonstrate that ASMase recruits palmitoylated CD36, but non-palmitoylated CD36 to form a complex in the membrane.

### ASMase deficiency reduces plaque CD36 LR recruitment and aortic lipid accumulation

**Figure 6:**
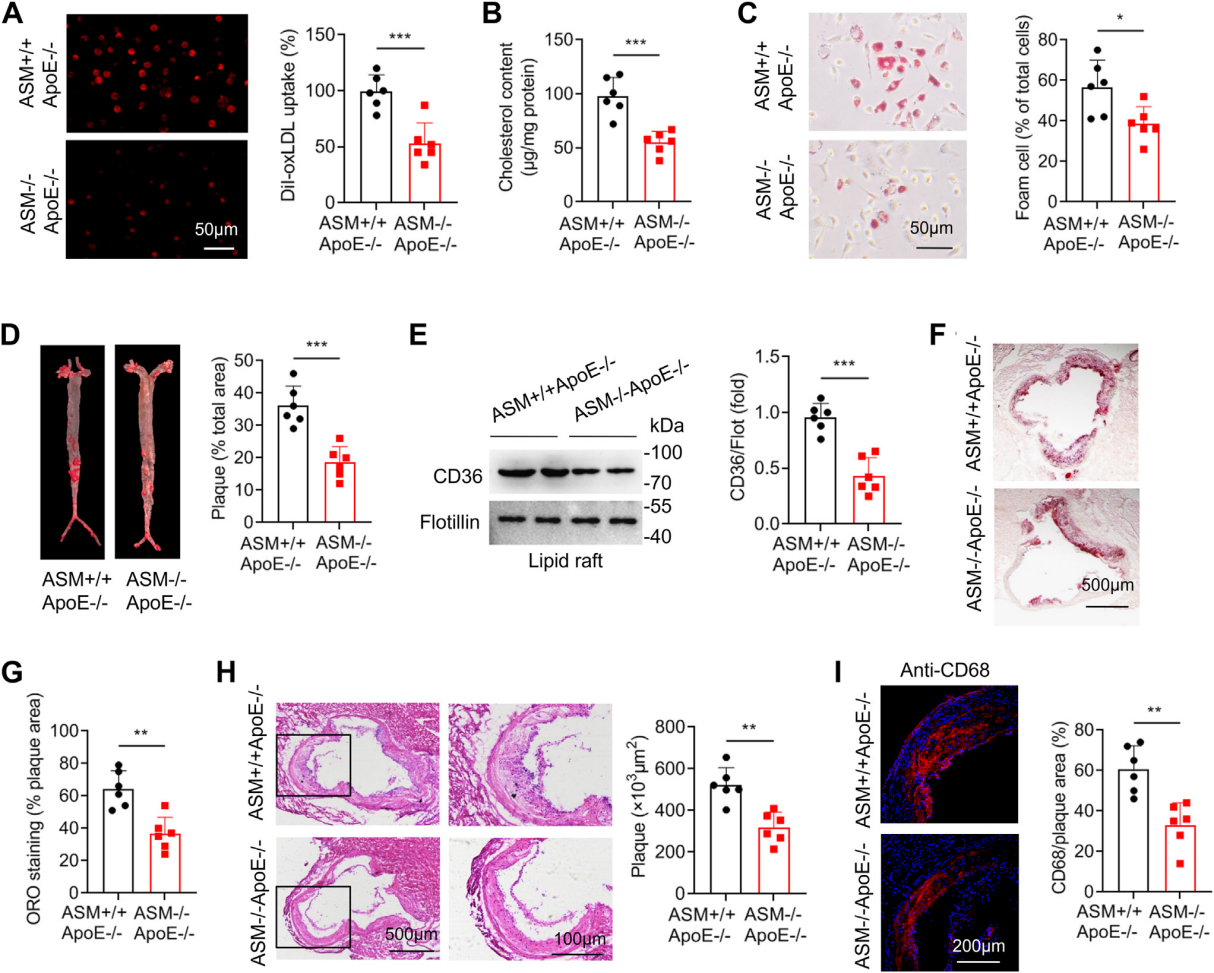
**ASMase deficiency reduces CD36 LR recruitment and aortic lipid accumulation in atherosclerotic plaques** ASM+/+ApoE−/− and ASM−/−ApoE−/− mice are fed a high-fat diet beginning at 8 weeks of age for 16. *A*, peritoneal macrophages isolated from ASM+/+ApoE−/− and ASM−/−ApoE−/− mice are incubated with DiI-oxLDL for 6 h, and DiI-oxLDL uptake is measured by flow cytometry. *B*, intracellular cholesterol levels in peritoneal macrophages are quantified. *C*, peritoneal macrophages are incubated with oxLDL for 24 h and stained with Oil Red O. *D*, whole aorta from ASM+/+ApoE−/− and ASM−/−ApoE−/− mice are stained with Oil Red O to assess plaque formation. *E*, CD36 LR localization is analyzed in macrophages isolated from high-fat diet ASM+/+ApoE−/− and ASM−/−ApoE−/− mice. *F* and *G*, Oil Red O staining of aortic root sections is performed in ASM+/+ApoE−/− and ASM−/−ApoE−/− mice. Positive staining in total plaque area was calculated. *H* and *I*, sections of the aortic roots are stained with HE and anti-CD68 antibodies. All data are analyzed by Student's *t* test, mean ± SD, n = 6. ∗*p* < 0.05, ∗∗*p* < 0.01, ∗∗∗*p* < 0.001.

ASM+/+ApoE−/− and double-deficient ASM−/−ApoE−/− mice were fed a HFD for 16 weeks. We assayed lipoprotein profile and apolipoprotein and found ASMase depletion significantly increased levels in triglyceride, LDL-C, and ApoB100 (Table S1). Primary peritoneal macrophages were isolated and incubated with DiI-oxLDL. ASMase deletion reduced DiI-oxLDL uptake (Fig. 6*A*) and decreased lipid accumulation and foam cell formation in macrophages incubated with oxLDL as assessed by Oil Red O staining (Fig. 6, *B* and *C*). Examination of the entire aorta revealed significantly fewer atherosclerotic plaques in ASMase-deficient mice (Fig. 6*D*). Proteins extracted from the aortic roots of ASM+/+ApoE−/− and ASM−/−ApoE−/− mice and LRs were isolated by ultracentrifugation. We found CD36 LR localization was reduced by 55% in ASM−/−ApoE−/− mice compared to that in ASM+/+ApoE−/− mice (Fig. 6*E*). Consequently, lipid deposits in the aortic root plaques were substantially reduced in ASM−/−ApoE−/− mice (Fig. 6, *F* and *G*). Hematoxylin and eosin (HE) staining revealed that ASMase deficiency attenuated atherosclerotic lesions evidenced by reducing plaque areas (Fig. 6*H*). Moreover, the aortic root sections were stained with macrophage marker, CD68 and results showed a significant reduction of intra-plaque macrophages in mice lacking ASMase (Fig. 6*I*). These findings suggest that ASMase deletion reduces aortic CD36 LR recruitment and lipid accumulation.

### OxLDL promotes SM catabolism

**Figure 7:**
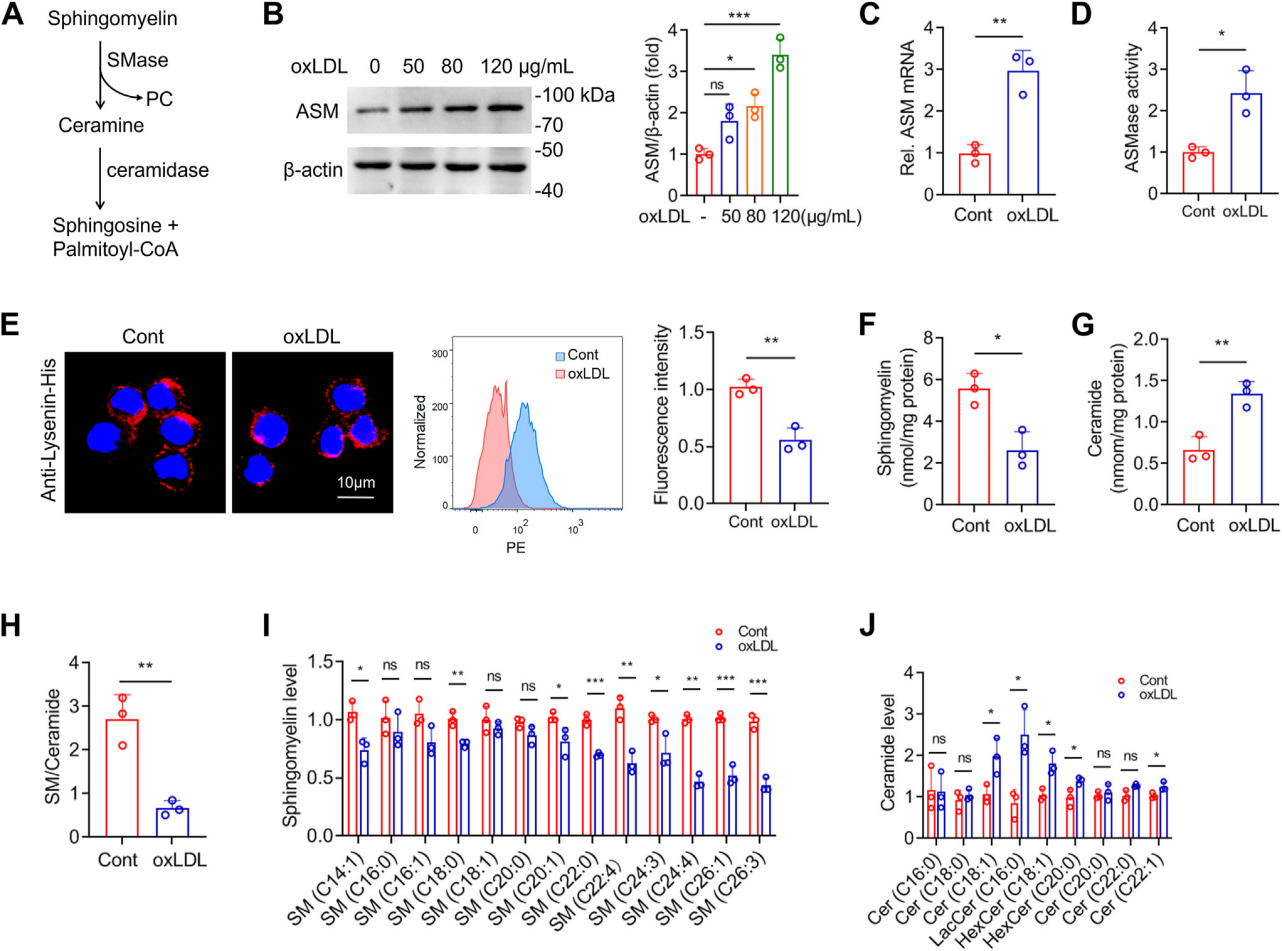
**OxLDL increases ASMase expression and promotes sphingomyelin (SM) catabolism.***A*, schematic illustration of SM catabolism. SM is hydrolyzed by ASMase to generate ceramide and phosphatidylcholine. Ceramidases convert ceramides into sphingosine and fatty acids. *B*, Western blot detection of ASMase expression is performed in cells treated with varying concentrations of oxLDL (50–120 μg/ml; one-way ANOVA followed by Tukey’s *post hoc* test, n = 3). *C*, RT-qPCR analysis measures ASMase mRNA levels in oxLDL (50 μg/ml) treated macrophages. *D*, ASMase enzymatic activity is assessed in cells treated with oxLDL at concentration of 50 μg/ml. *E*, OxLDL-treated cells are seeded on coverslip and incubated with SM-binding protein, lysenin-His (1 μg/ml). Membrane SM is detected using immunofluorescence staining and quantified by flow cytometry. *F*–*H*, total cellular levels of SM and ceramides, and SM/ceramide ratio are measured by LC-MS/MS in oxLDL-treated cells (50 μg/ml). *I* and *J*, lipidomics analysis of specific SM and ceramide species in cells treated with oxLDL (50 μg/ml). All comparisons were conducted with Student's *t* test except in (*B*) (mean ± SD, n = 3). ∗*p* < 0.05, ∗∗*p* < 0.01, ∗∗∗*p* < 0.001. ns, not significant.

Given that SM is the main substrate of ASMase (Fig. 7*A*), we investigated whether SM catabolism promoted palmitoylated CD36 LR recruitment. RAW264.7 cells incubated with various concentrations of oxLDL exhibited increased ASMase expression (Fig. 7, *B* and *C*). OxLDL also induced ASMase activity in the macrophages (Fig. 7*D*). Lysenin, a pore-forming toxin that binds specifically to SM, was used as an SM-specific probe (19). Lysenin labeling revealed that membrane SM levels were reduced upon oxLDL exposure (Fig. 7*E*). Moreover, reduced cellular SM levels and increased ceramide levels, thus reducing SM/ceramide ratio were detected in oxLDL-treated cells (Fig. 7, *F*–*H*). Lipidomic analysis by LS-MS/MS showed a decrease in various species of SM and ceramides, such as Cer(18:1) and modified ceramides, after oxLDL incubation (Fig. 7, *I* and *J*). These data suggest that oxLDL promotes SM catabolism.

### OxLDL upregulates ASMase by activating extracellular signal-regulated kinases/specificity protein 1 (ERKs/SP-1) signaling

**Figure 8:**
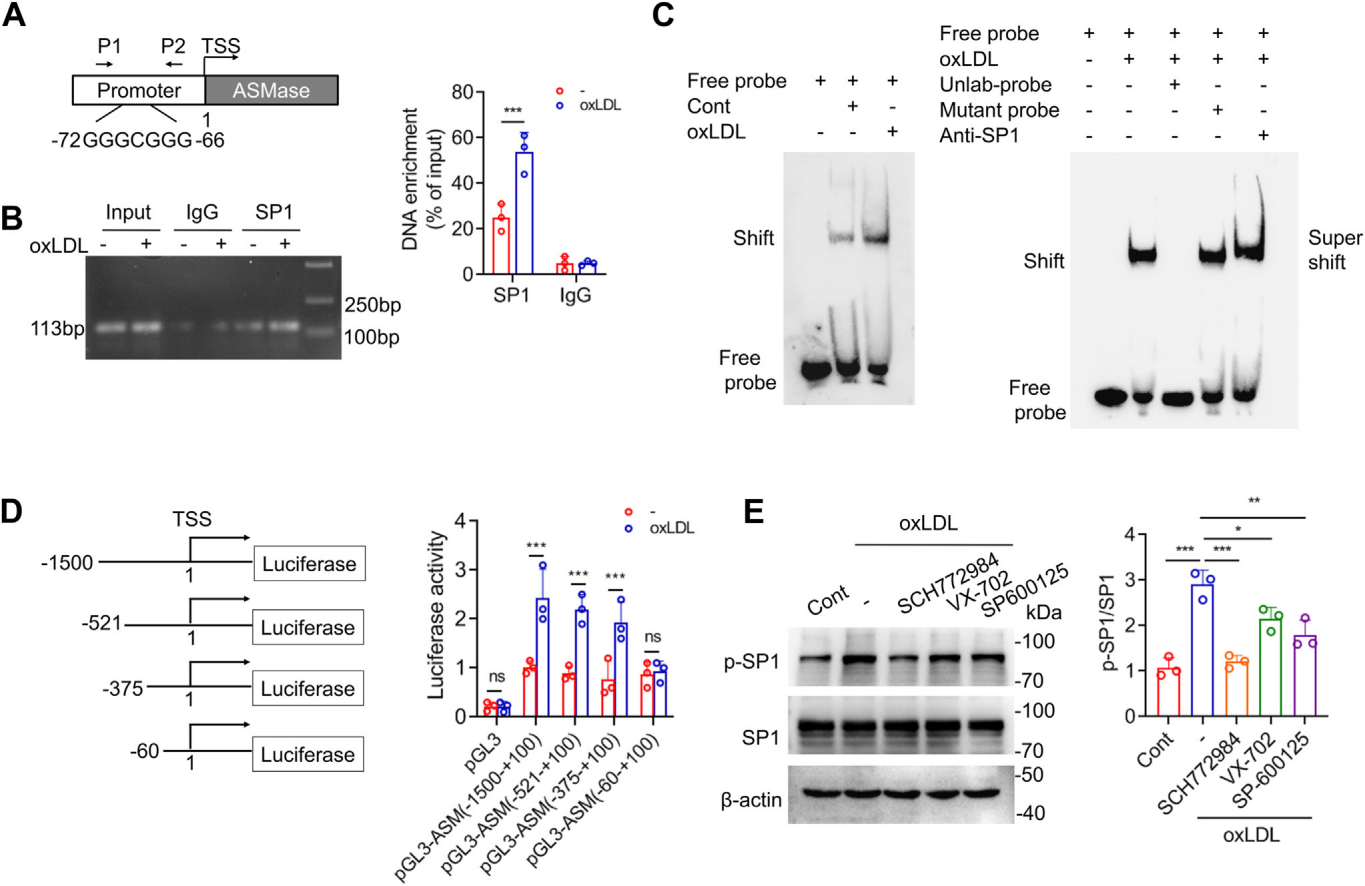
**ASMase is upregulated by activating ERKs/Sp-1 signaling in oxLDL-induced macrophages.***A*, schematic illustration shows the ASMase promoter containing an SP-1 binding site. Primers P1 and P2 are used for the ChIP assay. The primer sequences used are listed in the Experimental procedures section. *B*, the ChIP assay detects SP-1 binding to the ASMase promoter (two-way ANOVA followed by Tukey’s *post hoc* test). *C*, EMSA detects SP-1/ASMase promoter binding. *D*, luciferase reporter assay of truncated ASMase promoter activity. Student's *t* test. *E*, Western blot analysis measures SP-1 phosphorylation. Macrophages are treated with oxLDL and incubated with SCH772984, VX-702, or SP600125, which are specific inhibitors of ERK1/2, p38, and JNK1/2, respectively (one-way ANOVA followed by Tukey’s *post hoc* test). ∗*p* < 0.05, ∗∗*p* < 0.01, ∗∗∗*p* < 0.001. ns, not significant, mean ± SD, n = 3.

To further explore the signaling pathways and transcription factors involved in oxLDL-induced ASMase upregulation, we examined a conserved SP-1 recognition site located at −72 to −66 bp in the *Smpd1* promoter, which regulates ASMase transcription (Fig. 8*A*) (20). RAW264.7 cells incubated with oxLDL were subjected to ChIP analysis, revealing an increased binding of SP-1 to the *Smpd1* promoter (Fig. 8*B*). The increased *in vitro* binding induced by oxLDL was further demonstrated using EMSA (Fig. 8*C*). Deletion of the SP-1 recognition site of the promoter significantly reduced transcriptional activity, as shown by luciferase assay results (Fig. 8*D*). Finally, the mitogen-activated protein kinases, ERK1/2, p38, and JNK1/2 were inhibited using SCH772984, VX-702 and SP600125, respectively. SCH772984 treatment caused the most significant reduction in oxLDL-induced SP-1 phosphorylation (Fig. 8*E*). Therefore, the evidence indicates that oxLDL promotes ASMase expression through ERK/SP-1 activation.

## Discussion

**Figure 9:**
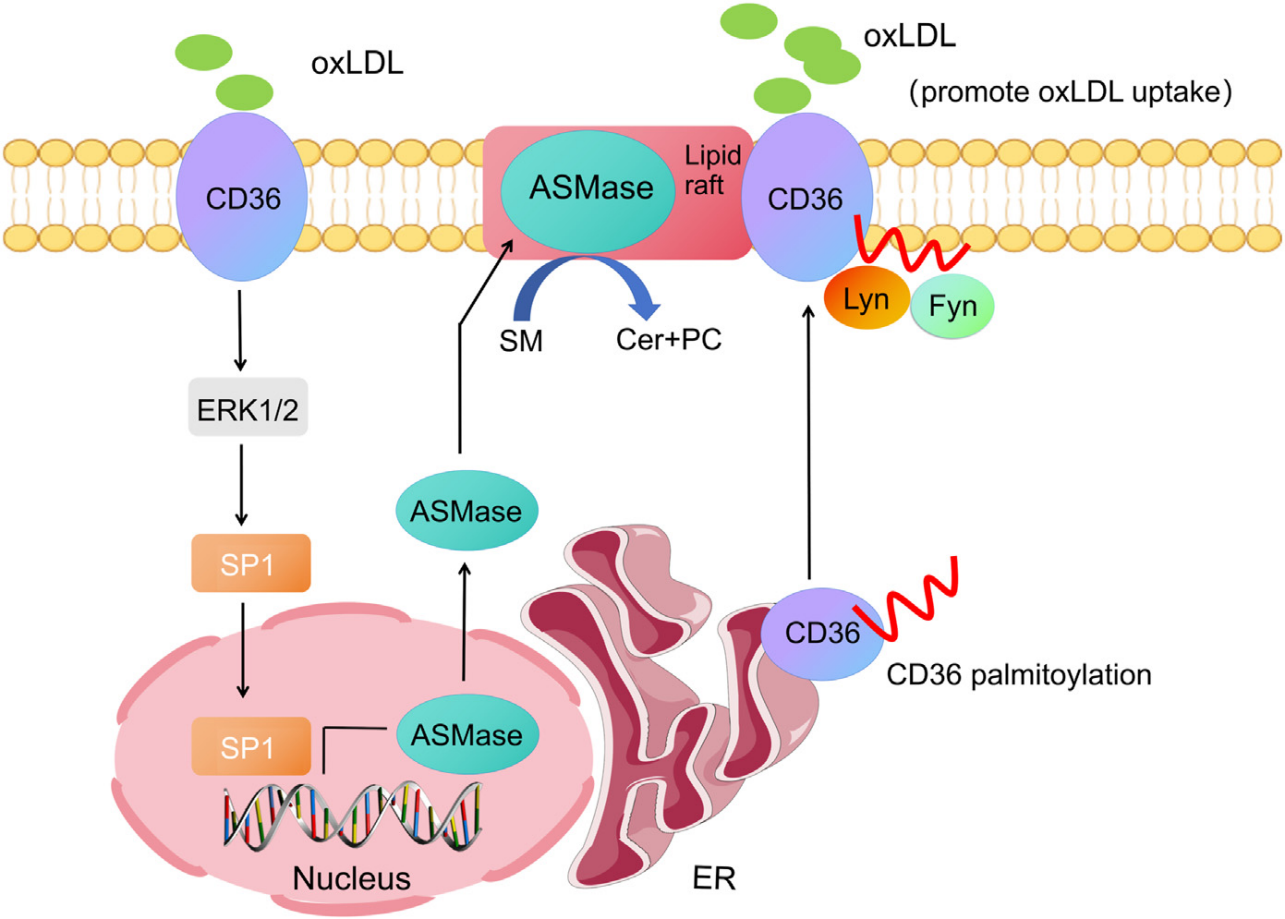
**Model for ASMase-mediated palmitoylated CD36 membrane rafts recruitment**.

Here, we provided evidence that oxLDL induces CD36 palmitoylation and membrane LR translocation, where CD36 interacts with ASMase. Moreover, oxLDL upregulates ASMase by activating ERK/Sp-1 signaling and promotes SM catabolism, facilitating palmitoylated CD36 membrane recruitment and increasing CD36 activity. These findings identify a novel mechanism underlying CD36-mediated foam cell formation by macrophages (Fig. 9).

Increased ASMase activity is observed in aging mice and in patients with acute coronary syndromes, and is considered a pro-atherogenic factor (21, 22). ASMase, generated and secreted by macrophages and vascular endothelial cells, profoundly affects foam cell formation and atherogenesis (23). ASMase is involved in foam cell formation through two mechanisms. First, extracellular ASMase catalyzes the hydrolysis of the lipoprotein SM, causing LDL aggregation and retention at the arterial wall and subendothelium (24), which triggers an inflammatory response and facilitates macrophage uptake. Second, intracellular sphingomyelinase enhances the binding of acyl coenzyme A-cholesterol acyltransferase to the LDL surface and increases enzymatic activity, which promotes cholesterol esterification and intracellular accumulation of cholesterol esters (9, 25). Our findings are consistent with those of previous studies showing that sphingomyelinase increases lipid uptake by macrophages. We further revealed that ASMase-induced oxLDL uptake depends on palmitoylation-mediated CD36 membrane translocation.

Protein palmitoylation, a conserved post-translational modification, involves the attachment of palmitate (C16:0) to a cysteine residue *via* a labile thioester bond. This modification profoundly affects protein function by modulating protein localization, trafficking, stability, and interactions (26). Among these, protein membrane translocation has a prominent effect owing to increased hydrophobicity and membrane affinity following protein modification. Membrane recruitment of palmitoylated proteins is involved in various pathophysiological processes (27, 28, 29). For instance, palmitoylation of lectin-like oxLDL receptor-1 (LOX-1) targets it to raft microdomains, enhancing its ligand uptake ability in human coronary artery endothelial cells (27). Palmitoylated gasdermin D (GSDMD) and nucleotide oligomerization domain-like receptor protein 3 (NLRP3) drive membrane translocation, which is critical for macrophage pyroptosis and inflammasome activation, respectively (28, 29). ASMase after translocation from the lysosome to the membrane triggers membrane fusion and LR clustering; the amplified membrane LRs providing a stable and large platform for molecular anchoring and assembly (30, 31, 32, 33). ASMase increases the plasma membrane content of glucose transporter type 4 (GLUT-4), which was reduces by MβCD treatment, suggesting that membrane recruitment is dependent on LRs (34, 35). In addition, enhanced ASMase activity and translocation to membrane LRs are associated with the assembly of cell surface proteins, such as epidermal growth factor receptor (EGFR), Toll-like receptor 4, and channel proteins, including cystic fibrosis transmembrane conductance regulator (CFTR) and transient receptor potential cation channel subfamily C 6 (TRPC6), a non-selective cation channel responsible for calcium and sodium influx (36, 37, 38, 39, 40). GLUT-4, EGFR, and CFTR undergo palmitoylation under various stimuli (41, 42, 43). Moreover, proteomic analyses have demonstrated that ASMase is associated with over 100 membrane proteins, including SNAP23, Src-family kinases Yes and Lyn, and Ras-, Rab-family small GTPases, most of which are palmitoylated (15). We reported that ASMase promotes CD36 membrane recruitment following palmitoylation. Therefore, whether ASMase-associated proteins, such as TRPC6, are palmitoylated, and whether ASMase affects palmitoylated proteins, such as LOX-1, GSDMD, and NLRP3, during their subcellular trafficking, are interesting topics for further research. Furthermore, it is conceivable that ASMase-dependent membrane translocation provides a general mechanism for the subcellular localization of palmitoylated proteins.

SM functions as a structural component of the plasma membrane and becomes a bioactive lipid after hydrolysis into ceramide and phosphatidylcholine by ASMase. We demonstrated that oxLDL increases ASMase activity and expression, resulting in a significant reduction in various SM species (Fig. 7). The levels of several ceramide species were expectedly increased after oxLDL stimulation. However, some ceramide species, such as Cer(C16:0), Cer(C20:1), Cer(C22:1), and Cer(24:1), were not significantly changed or even reduced in oxLDL-treated macrophages. A previous research has demonstrated that oxLDL increases the activity of ceramidases, enzymes responsible for ceramide catabolism, and elevates the cellular metabolism of sphingosine and S1P in cultured vascular smooth muscle cells (44). We confirmed these finding and observed increased ceramidase expression in macrophages following oxLDL treatment (data not shown). Therefore, we speculate that ceramide yield from SM hydrolysis may be rapidly reduced by increased ceramidase activity. On the other hand, the steady increase in PC levels further supports ASMase-mediated SM catabolism promoted by oxLDL.

Ceramide is central to sphingolipid metabolism, produced through *de novo* synthesis, the salvage pathway, and SM hydrolysis, and can be metabolized into other sphingolipids, such as sphingosine (Sph) and sphingosine 1-phosphate (S1P) (45). Ceramide is a highly active lipid that profoundly affects cellular biological process (1, 46). It promotes the clustering of SM-rich microdomains to form a ceramide-rich platform, which recruits membrane receptors (45). Ceramide can directly regulate downstream signaling molecules, such as MAPKs, nuclear factor-kappa B, and JAK, leading to apoptosis, cell cycle arrest, inflammation, and other cell pathophysiological changes (47, 48, 49, 50, 51, 52). Therefore, ceramides play a key role in the regulation of cellular signaling and cell fate. In this study, we demonstrated that CD36-mediated lipid uptake requires SM degradation. However, the exact mechanisms and role of ceramides in this process remain unclear and require further investigation.

In conclusion, we demonstrated that oxLDL induces ASMase expression and hydrolyzes membrane SM. This results in membrane LR clustering, facilitating the translocation of palmitoylated CD36 and increasing lipid uptake. Our works highlight the critical role of ASMase and provide novel insights into CD36-mediated foam cell formation and atherogenesis.

## Experimental procedures

### Reagents

ASM-N-1 was purchased from MCE . Ad-ASM, sh-ASM, mCD36 and mCD36 constructs were produced by GenePharma. Anti-CD36 (66395-1-Ig), anti-HA (51064-2-AP), Anti-Flag (20543-1-AP), Anti-ASM (14609-1-AP), Anti-Flotillin 1 (15571-1-AP), Anti-TfR (10084-2-AP) antibodies were from Proteintech. Anti-CD36 (ab252923), p-Jnk (ab124956) and anti-Caln (ab22595) antibodies were from Abcam. Lyn (#2732), Fyn (#4023) were from Cell Signaling Technology (CST). MβCD, N-Ethylmaleimide (NEM) and hydroxylamine (HAM) were purchased from Sigma-Aldrich. SCH772984, VX-702 and SP600125 were from Selleck (Houston, TX). The HA-tagged ASMase vector (HA-ASM) was generated by inserting *Smpd1* coding sequence at HindIII/BanHI site in pCMV-HA construct (Clontech) with HA tag at ASMase N-terminus. Recombinant acid sphingomyelinase protein (ab132844) was obtained from Abcam.

### Animals

ASMase deficient mice (ASM−/−) and apolipoprotein E deficiency mice (ApoE−/−) were purchased from GemPharmatech (Nanjing, China). The mice were maintained in a pathogen-free environment with 40–70% humidity, 22 °C ± 2 deg, and alternating 12 h light/dark cycle. ASM−/−ApoE−/− double knockout mice were generated by breeding. Male mice were fed a high-fat diet (HFD) beginning at 8 weeks of age for 16 weeks. After treatment, the mice were anesthetized and euthanized using an intraperitoneal overdose of pentobarbital sodium (150 mg/kg). Whole aortas and hearts were collected for analysis. All experimental procedures were performed in accordance with the guidelines approved by the Ethics Committee for Animals at Guizhou Medical University (No. 2200030).

### Measurement of serum lipids and apolipoprotein

Total blood were collected and placed on ice for 1 h. After centrifugation at 3000 rpm for 10 min, the supernatant serum was harvested and subjected to lipid and apolipoprotein measurements. Levels in total cholesterol (TC), triglyceride (TG), LDL-C and HDL-C were determined by enzymatic kits all from Jiancheng Company, and ApoB100 content was detected by ELISA kit (Jiancheng). The experiments were conducted following manufacture’s guidelines.

### Cell culture and isolation of peritoneal macrophages

RAW264.7 macrophages obtained from the American Type Culture Collection (Rockville, MD, USA) were cultured in Dulbecco's Modified Eagle’s Medium (DMEM, Gibco) containing 10% fetal bovine serum (FBS; HyClone) at 37 °C in 5% CO_2_. Peritoneal macrophages were isolated by performing a peritoneal lavage with ice-cold PBS on mice 3 days after intraperitoneal injection of 2 ml of 3% Brewer’s thioglycollate broth. Peritoneal macrophages were collected and cultured in DMEM supplemented with 10% FBS at 37 °C in 5% CO_2_.

### CD36 palmitoylation assay

CD36 palmitoylation was detected by immunoprecipitation and acyl-biotin exchange method (53). Briefly, cell lysis was CD36-immunoprecipitated and then unmodified thioester bonds were blocked in lysis buffer with 10 mM NEM. The sample was incubated with 1 M HAM for 50 min at room temperature to cleave thioester bonds at palmitoylated cysteines. After wash in lysis buffer, the beads were incubated with 500 μl lysis buffer containing 1 μM biotin-BMCC (Thermo Fisher Scientific) with rotation to selectively label the palmitoylated cysteines. The resulting thiol-biotinylated proteins were further detected with streptavidin–horseradish peroxidase (Beyotime) by Western blotting.

### Uptake of oxLDL and foam cell analysis

RAW264.7 and peritoneal macrophages were seeded in 24-well plates and treated with fluorescent 1,1' -dioctadecyl - 3,3,3′,3′-tetramethyl-indocarbocyanine perchlorate labeled oxLDL (Dil-oxLDL; 20 μg/ml; Yiyuan Biotechnology) for 6 h. DiI-oxLDL uptake was detected by fluorescence microscopy and quantified using flow cytometry (BD Biosciences). Data are expressed as the mean fluorescence intensity (MFI) acquired from at least 10,000 cells. For foam cell detection, cells were incubated with oxLDL (50 μg/ml, Yiyuan Biotechnology) for 24 h and stained with Oil Red O. Intracellular lipid accumulation was observed under a bright-field microscope.

### Immunofluorescence staining

Cells were cultured on coverslips and fixed with 4% paraformaldehyde for 15 min at room temperature. After blocking with 5% bovine serum albumin (BSA), the cells were incubated with primary antibodies overnight at 4 °C. The cells were then washed with phosphate-buffered saline (PBS) and incubated with fluorescein isothiocyanate (FITC)- or rhodamine-conjugated secondary antibodies (Proteintech), for 2 h at room temperature. Nuclei were counterstained with 4′,6-diamidino-2-phenylindole (DAPI, Beyotime). Glass coverslips with attached cells were mounted on microslides. Staining was visualized by confocal microscopy using a SpinSR10 microscope (Olympus).

### LR isolation

Membrane LRs were isolated using discontinuous sucrose gradient ultracentrifugation. Briefly, cells were lysed in 2 ml of ice-cold RIPA lysis buffer (Beyotime). The lysate was added to 2 ml of ice-cold 4-morpholine ethanesulfonic acid (MES)-buffered saline (25 mM MBS, pH 6.5, 150 mM NaCl) containing 80% sucrose. The samples were subsequently covered with 4 ml of 35% and 5% sucrose in MBS, followed by centrifugation for 16 h at 180,000*g* and 4 °C using a SW41 rotor (Beckman Coulter). Twelve fractions were collected from the top of the gradient for Western blot analysis.

### Co-immunoprecipitation and Western blotting

The cells were washed and lysed in RIPA lysis buffer (Beyotime). The cell lysates were incubated with protein A/G agarose beads and primary antibody overnight at 4 °C with constant rotation. The precipitated complexes were washed three times with PBS. Proteins bound to the beads were eluted by boiling in loading buffer and subjected to sodium dodecyl sulfate-polyacrylamide gel electrophoresis (SDS-PAGE). For Western blotting, the cells were lysed in RIPA lysis buffer (Beyotime). The proteins were resolved by SDS-PAGE and transferred to polyvinylidene difluoride membranes (Millipore). After blocking, the membranes were incubated with the specific antibodies overnight at 4 °C, followed by incubation with horseradish peroxidase (HRP)-conjugated secondary antibodies for 2 h at room temperature. The membranes were washed three times with Tris-buffered saline (TBS) containing 0.1% Tween-20 and visualized by enhanced chemiluminescence detection kit (Beyotime). Band densities were quantified using ImageJ software (NIH).

### Immunopurification and mass spectrometry

Cells were incubated with oxLDL and transfected with Flag-CD36. Membrane proteins were isolated, and CD36-bound proteins were purified by incubation with anti-Flag affinity gel (Beyotime) according to the instructions provided by the manufacturer at 4 °C overnight. After elution with TBS containing 3× Flag peptide, the precipitates were subjected to liquid chromatography tandem mass spectrometry (LC‒ MS/MS).

### GST-pulldown assay

GST-CD36 and HA-ASM expressing in BL21 cells were purified by using GST-tag purification resin (Beyotime) and anti-Flag affinity gel (Beyotime), respectively. For binding assay, GST-CD36 was incubated with Flag-ASM or cell lysates in GST pulldown binding buffer (50 mM Tris-HCl, 200 mM NaCl, 1 mM EDTA, 1% NP-40, 1 mM DTT, 10 mM MgCl_2_, pH 8.0) containing GST resin for 12 h at 4 °C. Bound proteins were run on SDS-PAGE and subjected to immunoblot.

### Chromatin immunoprecipitation (ChIP) assay and reverse transcription quantitative polymerase chain reaction (RT-qPCR)

ChIP assays were performed according to the instructions provided by the manufacturer (Beyotime). Cells were crosslinked for 10 min with 1% formaldehyde and lysed in 300 μl SDS lysis buffer containing 1 mM phenylmethylsulfonyl fluoride. Chromatin was sheared by sonication and immunoprecipitated overnight at 4 °C with anti-Sp-1 antibody following blocking with Protein A + G Agarose/Salmon Sperm DNA. The precipitated DNA was purified and analyzed by PCR/2% agarose gel or real-time PCR analysis using primers (Forward:5′-CGGGGTGATTGGGCTGTGGCT-3′; Reverse: 5′-AACGGGGCCCAGCAACTGTG-3′) specific to the SP1-binding site on the ASMase promoter. Before immunoprecipitation, DNA was used as an input control. The data are presented as changes related to the input.

For RT-qPCR, total cellular RNA was extracted using TRNzol Reagent (Tiangen). One microgram of RNA was reverse transcribed to complementary DNA (cDNA) using GoScript Reverse Transcription Mix (Promega). qPCR was performed using GoTaq qPCR Master Mix (Promega) on a CFX Duet PCR thermocycler (Bio-Rad). Data were normalized to glyceraldehyde 3-phosphate dehydrogenase and fold changes in mRNA levels were calculated using the 2^−ΔΔCt^ method.

### Electrophoretic mobility shift assay (EMSA)

DNA oligo from the ASMase promoter containing the Sp-1 binding site (5′-aggcggatcagggcgggttggcgagccc-3′) was biotin-labeled by terminal deoxynucleotidyl transferase and incubated with 10 μg of nuclear extracts prepared from oxLDL-treated RAW264.7 macrophages for 20 min at room temperature. Competitive binding assays were performed using 200-fold molar excesses of unlabeled or mutant probe, and supershift assays were carried out using 1 μg of anti-SP1 antibody (Abcam). Bound complexes were separated on a 6% non-denaturing PAGE gel in 0.5× Tris-borate-EDTA buffer and then transferred to hybond-N+ nylon membranes (GE Healthcare). After ultraviolet crosslinking, the membranes were incubated with streptavidin-conjugated HRP, and immunostaining was performed using the chemiluminescence method.

### Luciferase reporter assay

RAW264.7 cells were co-transfected with an ASMase promoter-driven firefly luciferase construct (pGL3-MMP2, Promega) and a Renilla luciferase-expressing plasmid (pRL-TK, Promega). After incubation with oxLDL for 24 h, the cells were harvested and luciferase activity was measured using a Dual Luciferase Reporter Gene Assay Kit (Beyotime) on a luminometer (Bio-Tek Instruments, Inc), according to the protocol provided by the manufacturer. Luciferase activity was determined in triplicate and normalized to Renilla luciferase activity.

### Histological analysis

For *en face* analysis, the entire aorta from the aortic arch to the iliac bifurcation was dissected, and the perivascular adipose tissues were removed. After staining with 0.3% Oil Red O, the aorta was cut open longitudinally to expose the lesions. The plaque was calculated as the proportion of positively stained areas relative to the total luminal surface. For analysis of lesions in aortic root, the upper part of heart was embedded in OTC compound, and serial cryosections (6 μm thick) were cut through the aortic valve. Sections were stained with Oil Red O and HE.

### Lysenin staining

Cells were grown on coverslips and fixed with 4% paraformaldehyde at room temperature. After blocking with 2% BSA/PBS, 1 μg/ml lysenin-His (TargetMol) dissolved in the blocking solution was added and incubated at 4 °C overnight. The cells were washed with PBS and incubated with anti-His antibody at 4 °C overnight. After washing, cells were incubated with rhodamine-conjugated goat anti-mouse IgG for 1 h at room temperature. Staining was visualized by confocal microscopy using the SpinSR10 microscope (Olympus), and quantified by flow cytometry (BD Biosciences).

### ASMase activity

ASMase activity was determined using an acid sphingomyelinase assay kit (ab252889, Abcam) according to the protocol given by the manufacturer. Briefly, after adding SM substrate, the reaction mixture was incubated for 30 min at 37 °C. The absorbance was measured at 570 nm and ASMase activity was calculated.

### Lipid extraction and LC-MS/MS analysis

Measurements of SM and ceramide was performed using LC-MS/MS. Cells were lysed by ultrasound, and the protein concentrations were determined by a BCA Protein Assay Kit (Beyotime). Lipids in the same total protein content were then extracted with 75% cold methyl alcohol and methyl tert-butyl ether and 10 μl EquiSPLASH (Avanti Research) containing SM and ceramide from Sigma-Aldrich used as internal standard on a rotational table at 4 °C for 1 h. After centrifugation at 16,000*g* for 10 min at 4 °C, the top organic phase layer was collected and completely dried under a nitrogen stream.

The lipid extracts were reconstituted in 200 μl of 10 mM ammonium acetate dichloromethane: methanol (50:50). Lipids were separated by ultra-high-performance LC using the Nexera LC-30AD system (Shimadzu). Solvent A consisted of 10 mM ammonium acetate and 50% acetonitrile (pH 8.0), and solvent B was 100% acetonitrile. The column temperature was maintained at 40 °C. The injection volume was 1 μl at 4 °C with a flow rate of 300 μl/min. The gradient profile was 85% B from 0 to 0.1 min, 65% B from 0.1 to 7.5 min, 5% B from 7.5 to 11 min, 85% B from 11 to 11.1 min, and 5% B from 11.1 to 15 min.

To quantify individual lipid species, samples were analyzed using a QTRAP5500 mass spectrometer (AB SCIEX) with both positive and negative electrospray ionization (ESI) modes. Data were processed using the LipidSearch software (ThermoFisher Scientific). Quality control samples were prepared by mixing equal aliquots of lipid extract of untreated and oxLDL-treated cells for assessing stability and repeatability of the analysis. The values for each lipid are presented as nmol/mg protein normalized to the untreated control cells.

### Statistical analyses

Data are expressed as mean ± standard deviation (SD) from at least three independent experiments. GraphPad Prism9 was used for statistical analysis. Comparisons between two groups were performed using two-tailed unpaired Student's t-tests. Multiple group comparisons were conducted using one-way analysis of variance (ANOVA), followed by Tukey’s *post hoc* test. Differences were considered statistically significant at *p* < 0.05.

## Data availability

All data supporting the findings of this study are available upon reasonable request.

## Supporting information

This article contains supporting information.

## Conflict of interest

The authors declare that they have no conflicts of interest with the contents of this article.

## References

[1] Adada M., Luberto C., Canals D. (2016). Inhibitors of the sphingomyelin cycle: sphingomyelin synthases and sphingomyelinases. Chem. Phys. Lipids.

[2] Breiden B., Sandhoff K. (2021). Acid sphingomyelinase, a lysosomal and secretory phospholipase C, is key for cellular phospholipid catabolism. Int. J. Mol. Sci..

[3] Head B.P., Patel H.H., Insel P.A. (2014). Interaction of membrane/lipid rafts with the cytoskeleton: impact on signaling and function: membrane/lipid rafts, mediators of cytoskeletal arrangement and cell signaling. Biochim. Biophys. Acta.

[4] Isik O.A., Cizmecioglu O. (2023). Rafting on the plasma membrane: lipid rafts in signaling and disease. Adv. Exp. Med. Biol..

[5] Capozzi A., Manganelli V., Riitano G., Caissutti D., Longo A., Garofalo T. (2023). Advances in the pathophysiology of thrombosis in antiphospholipid syndrome: molecular mechanisms and signaling through lipid rafts. J. Clin. Med..

[6] Jin S., Yi F., Zhang F., Poklis J.L., Li P.L. (2008). Lysosomal targeting and trafficking of acid sphingomyelinase to lipid raft platforms in coronary endothelial cells. Arterioscler. Thromb. Vasc. Biol..

[7] Ferranti C.S., Cheng J., Thompson C., Zhang J., Rotolo J.A., Buddaseth S. (2020). Fusion of lysosomes to plasma membrane initiates radiation-induced apoptosis. J. Cell Biol..

[8] Chistiakov D.A., Melnichenko A.A., Myasoedova V.A., Grechko A.V., Orekhov A.N. (2017). Mechanisms of foam cell formation in atherosclerosis. J. Mol. Med. (Berl).

[9] Xu X.X., Tabas I. (1991). Sphingomyelinase enhances low density lipoprotein uptake and ability to induce cholesteryl ester accumulation in macrophages. J. Biol. Chem..

[10] Shu H., Peng Y., Hang W., Nie J., Zhou N., Wang D.W. (2022). The role of CD36 in cardiovascular disease. Cardiovasc. Res..

[11] Zhang Y., Dong D., Xu X., He H., Zhu Y., Lei T. (2022). Oxidized high-density lipoprotein promotes CD36 palmitoylation and increases lipid uptake in macrophages. J. Biol. Chem..

[12] Eyre N.S., Cleland L.G., Mayrhofer G. (2008). FAT/CD36 expression alone is insufficient to enhance cellular uptake of oleate. Biochem. Biophys. Res. Commun..

[13] Meiler S., Baumer Y., Huang Z., Hoffmann F.W., Fredericks G.J., Rose A.H. (2013). Selenoprotein K is required for palmitoylation of CD36 in macrophages: implications in foam cell formation and atherogenesis. J. Leukoc. Biol..

[14] Rios F.J., Ferracini M., Pecenin M., Koga M.M., Wang Y., Ketelhuth D.F. (2013). Uptake of oxLDL and IL-10 production by macrophages requires PAFR and CD36 recruitment into the same lipid rafts. PLoS One.

[15] Xiong X., Lee C.F., Li W., Yu J., Zhu L., Kim Y. (2019). Acid Sphingomyelinase regulates the localization and trafficking of palmitoylated proteins. Biol. Open.

[16] Dias I.H., Mistry J., Fell S., Reis A., Spickett C.M., Polidori M.C. (2014). Oxidized LDL lipids increase β-amyloid production by SH-SY5Y cells through glutathione depletion and lipid raft formation. Free Radic. Biol. Med..

[17] Bao J.X., Jin S., Zhang F., Wang Z.C., Li N., Li P.L. (2010). Activation of membrane NADPH oxidase associated with lysosome-targeted acid sphingomyelinase in coronary endothelial cells. Antioxid. Redox Signal..

[18] Wähe A., Kasmapour B., Schmaderer C., Liebl D., Sandhoff K., Nykjaer A. (2010). Golgi-to-phagosome transport of acid sphingomyelinase and prosaposin is mediated by sortilin. J. Cell Sci..

[19] Shogomori H., Kobayashi T. (2008). Lysenin: a sphingomyelin specific pore-forming toxin. Biochim. Biophys. Acta.

[20] Langmann T., Buechler C., Ries S., Schaeffler A., Aslanidis C., Schuierer M. (1999). Transcription factors Sp1 and AP-2 mediate induction of acid sphingomyelinase during monocytic differentiation. J. Lipid Res..

[21] Kobayashi K., Nagata E., Sasaki K., Harada-Shiba M., Kojo S., Kikuzaki H. (2013). Increase in secretory sphingomyelinase activity and specific ceramides in the aorta of apolipoprotein E knockout mice during aging. Biol. Pharm. Bull..

[22] Pan W., Yu J., Shi R., Yan L., Yang T., Li Y. (2014). Elevation of ceramide and activation of secretory acid sphingomyelinase in patients with acute coronary syndromes. Coron. Artery Dis..

[23] Marathe S., Schissel S.L., Yellin M.J., Beatini N., Mintzer R., Williams K.J. (1998). Human vascular endothelial cells are a rich and regulatable source of secretory sphingomyelinase. Implications for early atherogenesis and ceramide-mediated cell signaling. J. Biol. Chem..

[24] Oörni K., Posio P., Ala-Korpela M., Jauhiainen M., Kovanen P.T. (2005). Sphingomyelinase induces aggregation and fusion of small very low-density lipoprotein and intermediate-density lipoprotein particles and increases their retention to human arterial proteoglycans. Arterioscler. Thromb. Vasc. Biol..

[25] Stein O., Ben-Naim M., Dabach Y., Hollander G., Stein Y. (1992). Modulation of sphingomyelinase-induced cholesterol esterification in fibroblasts, CaCo2 cells, macrophages and smooth muscle cells. Biochim. Biophys. Acta.

[26] Jin J., Zhi X., Wang X., Meng D. (2021). Protein palmitoylation and its pathophysiological relevance. J. Cell Physiol..

[27] Kumano-Kuramochi M., Xie Q., Kajiwara S., Komba S., Minowa T., Machida S. (2013). Lectin-like oxidized LDL receptor-1 is palmitoylated and internalizes ligands via caveolae/raft-dependent endocytosis. Biochem. Biophys. Res. Commun..

[28] Yu T., Hou D., Zhao J., Lu X., Greentree W.K., Zhao Q. (2024). NLRP3 Cys126 palmitoylation by ZDHHC7 promotes inflammasome activation. Cell Rep..

[29] Balasubramanian A., Hsu A.Y., Ghimire L., Tahir M., Devant P., Fontana P. (2024). The palmitoylation of gasdermin D directs its membrane translocation and pore formation during pyroptosis. Sci. Immunol..

[30] Koka S., Xia M., Chen Y., Bhat O.M., Yuan X., Boini K.M. (2017). Endothelial NLRP3 inflammasome activation and arterial neointima formation associated with acid sphingomyelinase during hypercholesterolemia. Redox Biol..

[31] Wei J., Xu L., Du Y.N., Tang X.F., Ye M.Q., Wu Y.J. (2019). Membrane raft redox signalling contributes to endothelial dysfunction and vascular remodelling of thoracic aorta in angiotensin II-infused rats. Exp. Physiol..

[32] Li X., Wang H.F., Li X.X., Xu M. (2019). Contribution of acid sphingomyelinase to angiotensin II-induced vascular adventitial remodeling via membrane rafts/Nox2 signal pathway. Life Sci..

[33] Yuan X., Bhat O.M., Zou Y., Li X., Zhang Y., Li P.L. (2022). Endothelial acid sphingomyelinase promotes NLRP3 inflammasome and neointima formation during hypercholesterolemia. J. Lipid Res..

[34] David T.S., Ortiz P.A., Smith T.R., Turinsky J. (1998). Sphingomyelinase has an insulin-like effect on glucose transporter translocation in adipocytes. Am. J. Physiol..

[35] Liu P., Leffler B.J., Weeks L.K., Chen G., Bouchard C.M., Strawbridge A.B. (2004). Sphingomyelinase activates GLUT4 translocation via a cholesterol-dependent mechanism. Am. J. Physiol. Cell Physiol..

[36] Tawadros P.S., Powers K.A., Ailenberg M., Birch S.E., Marshall J.C., Szaszi K. (2015). Oxidative stress increases surface toll-like receptor 4 expression in murine macrophages via ceramide generation. Shock.

[37] Wang Y., Li X., Sun L., Feng B., Sun W. (2016). Acid sphingomyelinase mediates 50-Hz magnetic field-induced EGF receptor clustering on lipid raft. J. Recept. Signal Transduct. Res..

[38] Zeitler S., Ye L., Andreyeva A., Schumacher F., Monti J., Nürnberg B. (2019). Acid sphingomyelinase - a regulator of canonical transient receptor potential channel 6 (TRPC6) activity. J. Neurochem..

[39] Abu-Arish A., Pandžić E., Kim D., Tseng H.W., Wiseman P.W., Hanrahan J.W. (2019). Agonists that stimulate secretion promote the recruitment of CFTR into membrane lipid microdomains. J. Gen. Physiol..

[40] Jiang T., Samapati R., Klassen S., Lei D., Erfinanda L., Jankowski V. (2022). Stimulation of the EP_3_ receptor causes lung oedema by activation of TRPC6 in pulmonary endothelial cells. Eur. Respir. J..

[41] Miura G.I., Buglino J., Alvarado D., Lemmon M.A., Resh M.D., Treisman J.E. (2006). Palmitoylation of the EGFR ligand Spitz by Rasp increases Spitz activity by restricting its diffusion. Dev. Cell.

[42] McClure M., DeLucas L.J., Wilson L., Ray M., Rowe S.M., Wu X. (2012). Purification of CFTR for mass spectrometry analysis: identification of palmitoylation and other post-translational modifications. Protein Eng. Des. Sel..

[43] Du K., Murakami S., Sun Y., Kilpatrick C.L., Luscher B. (2017). DHHC7 palmitoylates glucose transporter 4 (Glut4) and regulates Glut4 membrane translocation. J. Biol. Chem..

[44] Augé N., Nikolova-Karakashian M., Carpentier S., Parthasarathy S., Nègre-Salvayre A., Salvayre R. (1999). Role of sphingosine 1-phosphate in the mitogenesis induced by oxidized low density lipoprotein in smooth muscle cells via activation of sphingomyelinase, ceramidase, and sphingosine kinase. J. Biol. Chem..

[45] Taniguchi M., Okazaki T. (2021). Role of ceramide/sphingomyelin (SM) balance regulated through "SM cycle" in cancer. Cell Signal..

[46] Chakraborty M., Jiang X.C. (2013). Sphingomyelin and its role in cellular signaling. Adv. Exp. Med. Biol..

[47] Ragg S.J., Kaga S., Berg K.A., Ochi A. (1998). The mitogen-activated protein kinase pathway inhibits ceramide-induced terminal differentiation of a human monoblastic leukemia cell line, U937. J. Immunol..

[48] Sun X., Liu C., Qian M., Zhao Z., Guo J. (2010). Ceramide from sphingomyelin hydrolysis differentially mediates mitogen-activated protein kinases (MAPKs) activation following cerebral ischemia in rat hippocampal CA1 subregion. J. Biomed. Res..

[49] Lu S., Natarajan S.K., Mott J.L., Kharbanda K.K., Harrison-Findik D.D. (2016). Ceramide induces human hepcidin gene transcription through JAK/STAT3 pathway. PLoS One.

[50] Villasmil M.L., Francisco J., Gallo-Ebert C., Donigan M., Liu H.Y., Brower M. (2016). Ceramide signals for initiation of yeast mating-specific cell cycle arrest. Cell Cycle.

[51] Lallement J., Raho I., Merlen G., Rainteau D., Croyal M., Schiffano M. (2023). Hepatic deletion of serine palmitoyl transferase 2 impairs ceramide/sphingomyelin balance, bile acids homeostasis and leads to liver damage in mice. Biochim. Biophys. Acta Mol. Cell Biol. Lipids.

[52] Oliviero B., Dei Cas M., Zulueta A., Maiello R., Villa A., Martinelli C. (2023). Ceramide present in cholangiocarcinoma-derived extracellular vesicle induces a pro-inflammatory state in monocytes. Sci. Rep..

[53] Brigidi G.S., Bamji S.X. (2013). Detection of protein palmitoylation in cultured hippocampal neurons by immunoprecipitation and acyl-biotin exchange (ABE). J. Vis. Exp..

