# Supplementary material for: Acid sphingomyelinase recruits palmitoylated CD36 to membrane rafts and enhances lipid uptake
Source: J Biol Chem. 2025 May 8;301(6):110213. doi: 10.1016/j.jbc.2025.110213 (PMC12180984; doi:10.1016/j.jbc.2025.110213)
Supplement: Supporting information [file mmc1.docx]

**Supplementary material**

**Acid sphingomyelinase recruits palmitoylated CD36 to membrane rafts and enhances lipid uptake**

Meng Ding#, Yun Zhang#, Xiaoting Xu#, Yuan Zhu, Hui He, Tianyu Jiang, Yashuang Huang, Wenfeng Yu, Hailong Ou*

Department of Biochemistry and Molecular Biology, School of Basic Medicine, Guizhou Medical University, Gui'an 561113, Guizhou, China

#Meng Ding, Yun Zhang, and Xiaoting Xu: Contributed equally to this work

The file contains materials:

Table S1

Figures S1-S7

**Table S1. Serum lipids and body weight in ASM+/+ApoE-/- and ASM-/-ApoE-/- mice with HFD for 12 weeks.**

|  | TC  (mg/dl) | TG  (mg/dl) | LDL-C  (mg/dl) | HDL-C  (mg/dl) | ApoB  (mg/dl) | Body  weight (g) |
| --- | --- | --- | --- | --- | --- | --- |
| ASM+/+  ApoE-/- | 1175±  153.2 | 103.8±  17.1 | 811.2±  153.1 | 32.33±  6.408 | 21.12±  2.77 | 24.83±  2.238 |
| ASM-/-  ApoE-/- | 1328±  201.8^ns^ | 148.5±  24.7** | 1035±  121.8* | 26.83±  7.859^ns^ | 29.88±  6.304* | 23.08±  2.899^ns^ |

TC: total cholesterol, TG: triglyceride, LDL-C: low-density lipoprotein cholesterol, HDL-C: high-density lipoprotein cholesterol; HFD: high fat diet.

Data were expressed as mean ±SD and analyzed by Student’s t test (n=6), ns: non-significance, **p*<0.05,***p*<0.01, compared with ASM+/+ApoE-/- mice.

**
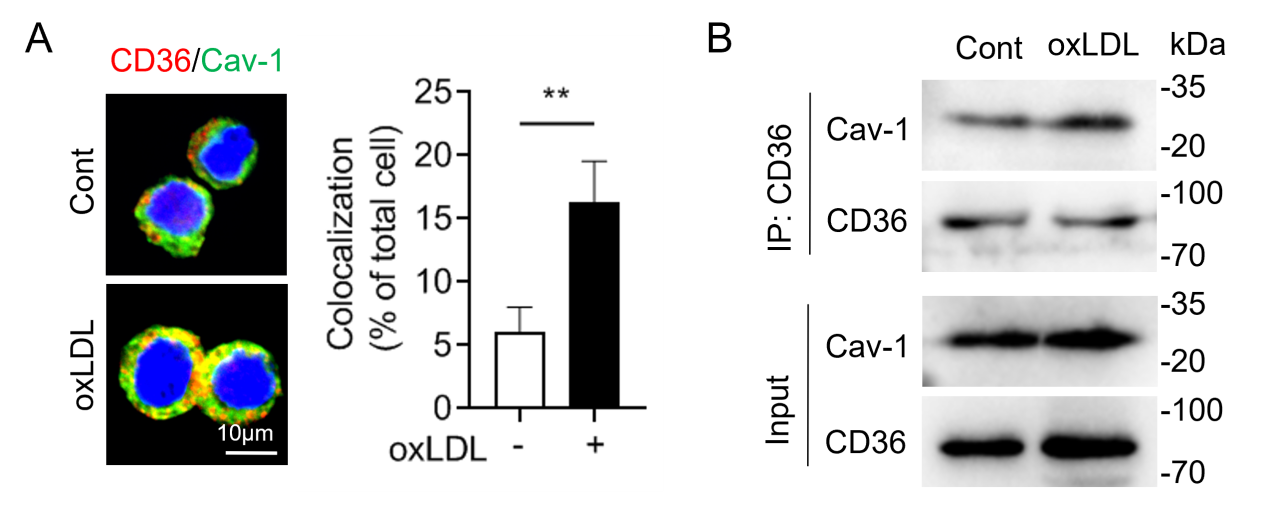
Fig. S1. OxLDL induces the association of CD36 and Cav-1 in RAW264.7 macrophages.**

RAW264.7 cells were incubated with oxLDL (50 μg/mL) for 24 h. (A) The cells were stained with anti-CD36 (red) and anti-Cav-1 (green) antibodies. The cells with the colocalization of CD36 and Cav-1 in total cells were counted. Student's t-test, mean ± SD, n=3. ***p*< 0.01. (B) Interaction of CD36 and Cav-1 was detected by co-immunoprecipitation.

**
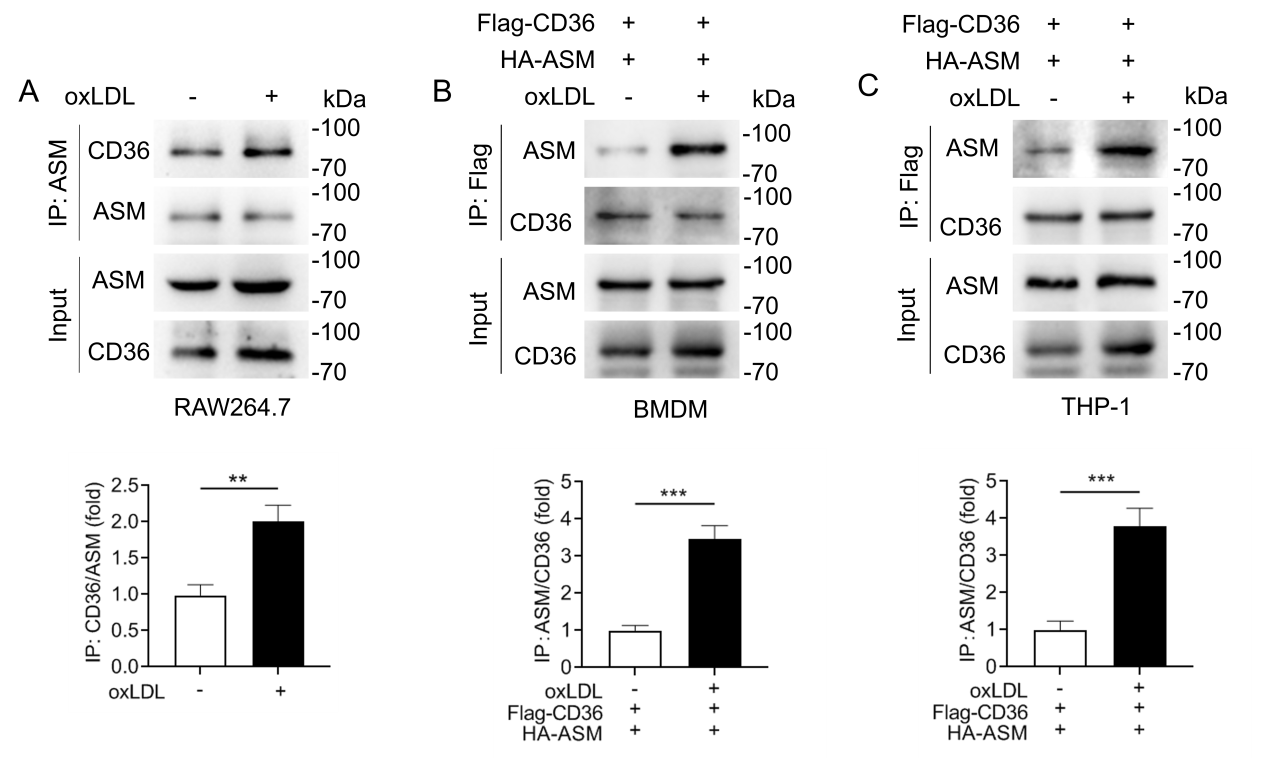
Fig. S2. CD36 associates with ASMase in oxLDL-treated macrophages.**

(A) Co-immunoprecipitation analysis of the association of CD36 and ASMase in RAW264.7 cells incubated with oxLDL (50 μg/mL) for 24 h. (B,C) BMDM and THP-1 cells were co-transfected with Flag-CD36 and HA-ASM followed by incubation with oxLDL for 24 h. CD36/ASMase complex was detected by co-immunoprecipitation. Student's t-test, mean ± SD, n=3. ***p*< 0.01, ****p*<0.001.

**
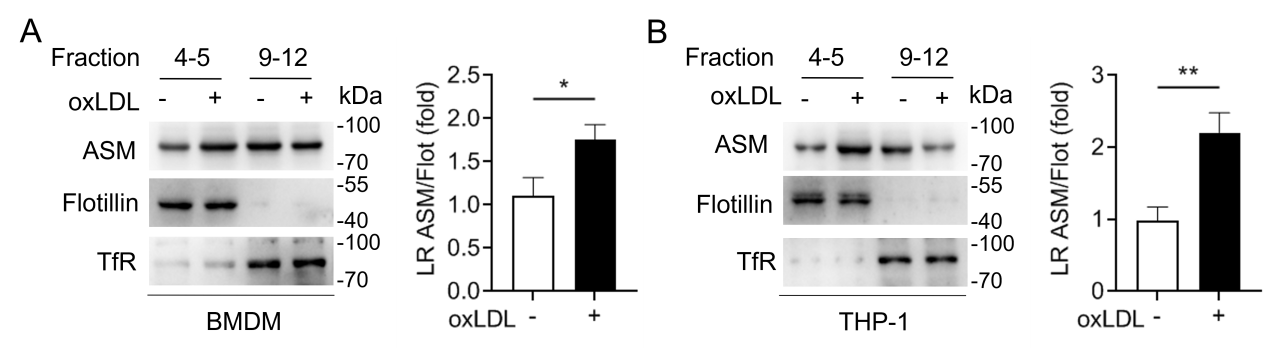
Fig. S3. Increased ASM LR translocation in oxLDL-treated BMDM and THP-1 cells.**

BMDM and THP-1 cells were incubated with oxLDL (50μg/mL) for 24 h, and the LR ASM in LR were detected by Western blot. Student's t-test, mean ± SD, n=3. **p*< 0.05, ***p*<0.01.

**
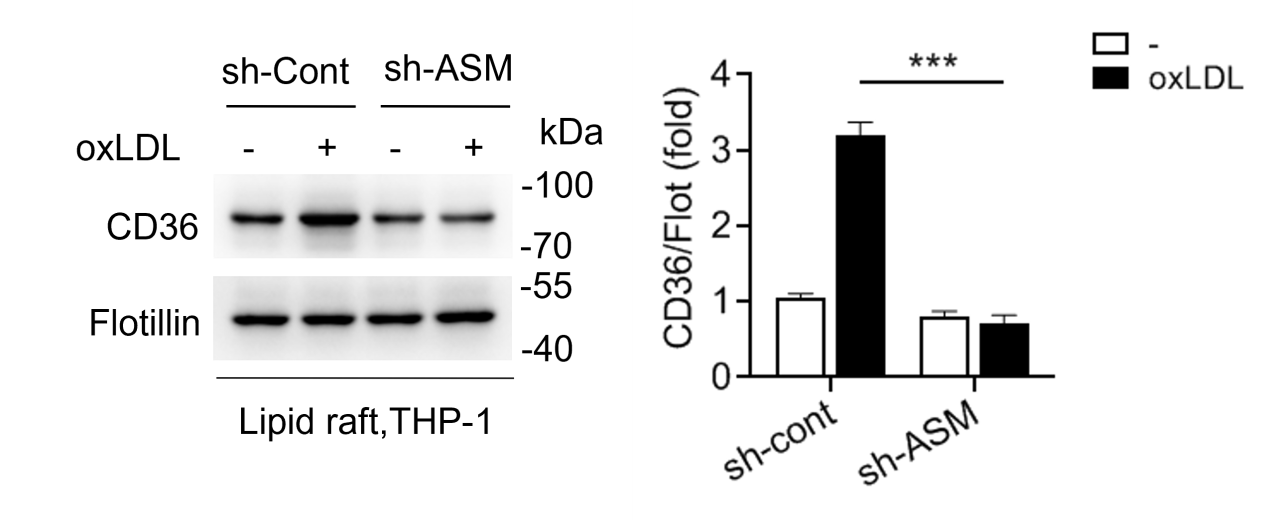
Fig. S4. ASMase knockdown reduces CD36 membrane LR localization in THP-1 cells.**

THP-1 cells were transfected with sh-ASMase and incubated with oxLDL for 24 h. LRs were isolated and CD36 in LRs was detected by Western blot. Two-way ANOVA test, mean ± SD, n=3. ****p*<0.001.

**
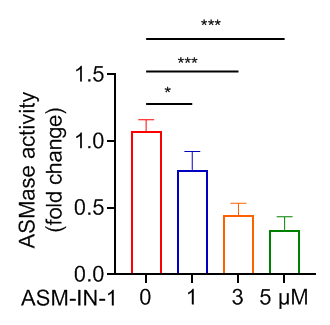
**

**Fig. S5. Effects of ASM-IN-1 on ASMase activity.**

RAW264.7 macrophages were incubated with different doges of ASMase inhibitor ASM-IN-1, and the ASMase activities were measured. One-way ANOVA test, mean ± SD, n=3. **p*<0.05. ****p*<0.001.


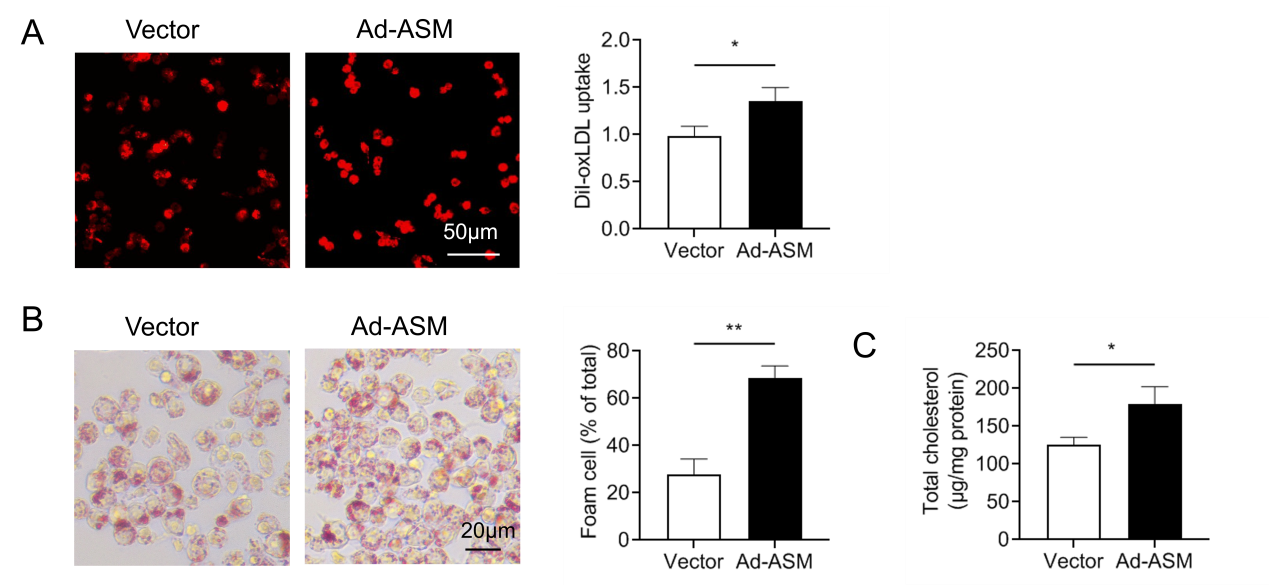


**Fig. S6. Effects of ASMase overexpression on oxLDL uptake, foam cell formation and intracellular cholesterol accumulation in RAW264.7 cells.**

(A) RAW264.7 cells were incubated with 30 μg/mL DiI-oxLDL for 6 h and observed under fluorescence microscope. The lipid uptake was quantified by fluorescence microplate reader. (B) RAW264.7 cells were incubated with oxLDL (50 μg/mL) for 24 h and stained with oil red O. (C) intracellular cholesterol accumulation in RAW264.7 cells treated with oxLDL (50 μg/mL) for 24 h. Student's t-test, mean ± SD, n=3. **p*<0.05, ***p*<0.01.

**
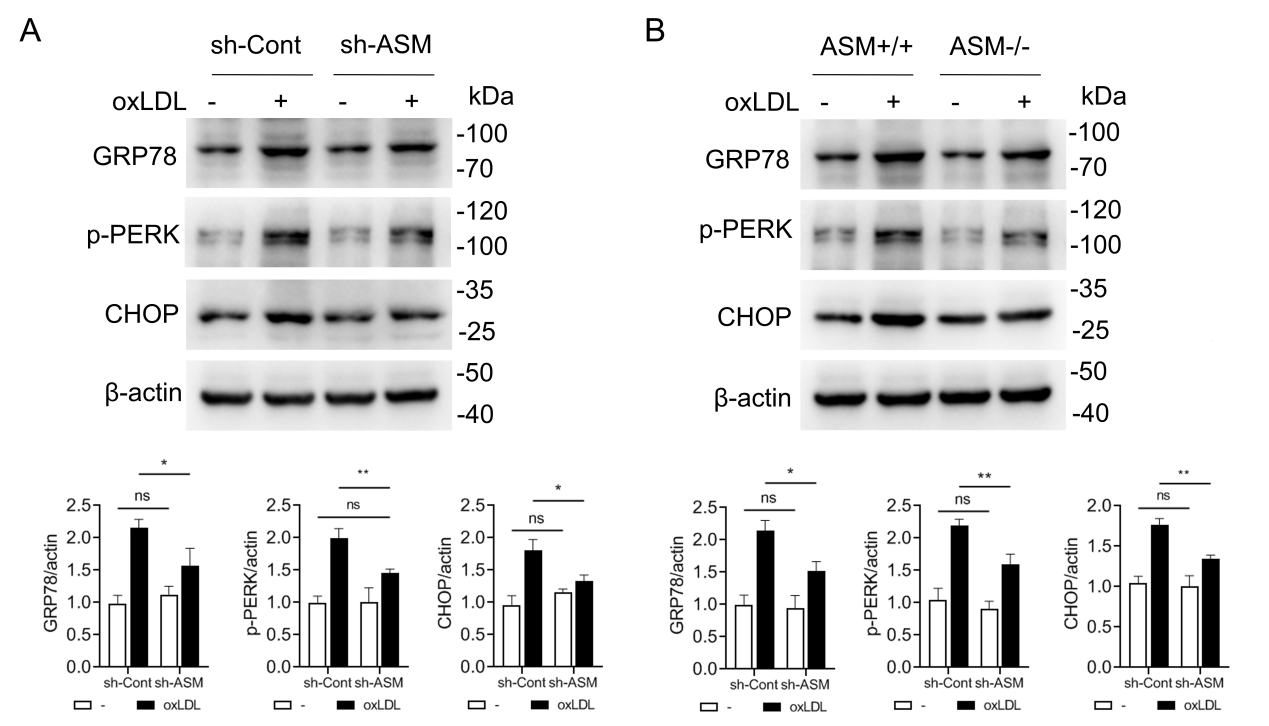
Fig. S7. Effects of ASMase knockdown on ER stress.**

(A) RAW264.7 macrophages were trasnfected with sh-ASM and were incubated with or without 50 μg/mL oxLDL for 24 h. The changes of ER stress markers were detected by Western blot. (B) Western blot analysis of indicated protein expression in ASM-/- macrophages incubated with or without 50 μg/mL oxLDL for 24 h. Two-way ANOVA test, mean ± SD, n=3, ns: non-significance, **p*<0.05, ***p*<0.01.
